# On Two Novel Parameters for Validation of Predictive QSAR Models

**DOI:** 10.3390/molecules14051660

**Published:** 2009-04-29

**Authors:** Partha Pratim Roy, Somnath Paul, Indrani Mitra, Kunal Roy

**Affiliations:** Drug Theoretics and Cheminformatics Lab, Division of Medicinal and Pharmaceutical Chemistry, Department of Pharmaceutical Technology, Jadavpur University, Kolkata 700 032, India; E-mails: partha_chemju@yahoo.co.in (P-P.R.), somnath_juph@yahoo.co.in (S.P.), indranimitra06@gmail.com (I.M.)

**Keywords:** QSAR, Validation, Internal validation, External validation, Randomization

## Abstract

Validation is a crucial aspect of quantitative structure–activity relationship (QSAR) modeling. The present paper shows that traditionally used validation parameters (leave-one-out Q^2^ for internal validation and predictive R^2^ for external validation) may be supplemented with two novel parameters r_m_^2^ and R_p_^2^ for a stricter test of validation. The parameter r_m_^2^_(overall)_ penalizes a model for large differences between observed and predicted values of the compounds of the whole set (considering both training and test sets) while the parameter R_p_^2^ penalizes model R^2^ for large differences between determination coefficient of nonrandom model and square of mean correlation coefficient of random models in case of a randomization test. Two other variants of r_m_^2^ parameter, r_m_^2^_(LOO)_ and r_m_^2^_(test)_, penalize a model more strictly than Q^2^ and R^2^_pred_ respectively. Three different data sets of moderate to large size have been used to develop multiple models in order to indicate the suitability of the novel parameters in QSAR studies. The results show that in many cases the developed models could satisfy the requirements of conventional parameters (Q^2^ and R^2^_pred_) but fail to achieve the required values for the novel parameters r_m_^2^ and R_p_^2^. Moreover, these parameters also help in identifying the best models from among a set of comparable models. Thus, a test for these two parameters is suggested to be a more stringent requirement than the traditional validation parameters to decide acceptability of a predictive QSAR model, especially when a regulatory decision is involved.

## 1. Introduction 

Quantitative structure-activity relationships (QSARs) are statistically derived models that can be used to predict the physicochemical and biological (including toxicological) properties of molecules from the knowledge of chemical structure. The structural features and properties are encoded within descriptors in numerical form. Descriptors support application of statistical tools generating relations which correlate activity data with descriptors (properties) in quantitative fashion. The description of QSAR models has been a topic for scientific research for more than 40 years and a topic within the regulatory framework for more than 20 years [1]. In the field of QSAR, the main objective is to investigate these relationships by building mathematical models that explain the relationship in a statistical way with ultimate goal of prediction and/or mechanistic interpretation. QSARs are being applied in many disciplines like drug discovery and lead optimization, risk assessment and toxicity prediction, regulatory decisions and agrochemicals [2,3,4]. One of the major applications of QSAR models is to predict the biological activity of untested compounds from their molecular structures [5]. The estimation of accuracy of predictions is a critical problem in QSAR modeling [6]. Only recently, validation of QSAR models has received considerable attention [7,8,9,10,11,12,13,14,15,16,17,18,19]. Four tools of assessing validity of QSAR models [20] are (i) randomization of the response data, (ii) cross-validation, (iii) bootstrapping, (iv) external validation by splitting of set of chemical compounds into a training and a test set and/or confirmation using an independent external validation set or external validation using a designed validation set. In order to be considered for regulatory use, especially in view of REACH (Registration, Evaluation, and Authorization of Chemicals) [1,21,22] legislation enforced in the European Union, it is widely agreed that QSARs need to be assessed in terms of their scientific validity, so that regulatory bodies have a sound scientific basis on which decisions regarding regulatory implementation can be taken. Several principles for assessing the validity of QSAR models were proposed at an International workshop held in Setubal (Portugal), which were subsequently modified in 2004 by the OECD Work Programme on QSARs [21,22]. Against this background, a review of the performance of the traditional validation parameters and the search for novel parameters which may be better metrics than the currently used ones appear to be of current need.

Recently the use of internal versus external validation has been a matter of great debate [23]. One group of QSAR workers supports internal validation, while the other group considers that internal validation is not a sufficient test for checking robustness of the models and external validation must be done. Hawkins *et al*., the major group of supporters of internal validation, are of the opinion that cross-validation is able to assess the model fit and to check whether the predictions will carry over to fresh data not used in the model fitting exercise. They have argued that when the sample size is small, holding a portion of it back for testing is wasteful and it is much better to use “computationally more burdensome” leave-one-out cross-validation [24,25].

An inconsistency between internal and external predictivity was reported in a few QSAR studies [26,27,28]. It was reported that, in general, there is no relationship between internal and external predictivity [29]: high internal predictivity may result in low external predictivity and *vice versa*.

Recently we have shown [15] that predictive R^2^ (R^2^_pred_) may not be a suitable measure to indicate external predictability, as it is highly dependent on training set mean. An alternative measure r_m_^2^ (based on observed and predicted data of the test set compounds) was suggested to be a better metric to indicate external predictability. But it can as well be applied for training set if one considers the correlation between observed and leave-one-out (LOO) predicted values of the training set compounds [30,31]. More interestingly, this can be used for the whole set considering LOO-predicted values for the training set and predicted values of the test set compounds. The advantages of such consideration are: (1) unlike external validation parameters (R^2^_pred_ etc.), the r_m_^2^_(overall)_ statistic is not based only on limited number of test set compounds. It includes prediction for both test set and training set (using LOO predictions) compounds. Thus, this statistic is based on prediction of comparably large number of compounds. In many cases, test set size is considerably small and regression based external validation parameter may be less reliable and highly dependent on individual test set observations. In such cases, the r_m_^2^_(overall)_ statistic may be advantageous. (2) In many cases, comparable models are obtained where some models show comparatively better internal validation parameters and some other models show comparatively superior external validation parameters. This may create a problem in selecting the final model. The r_m_^2^_(overall)_ statistic may be used for selection of the best predictive models from among comparable models.

Again, for an acceptable QSAR model, the average correlation coefficient (R_r_) of randomized models should be less than the correlation coefficient (R) of the non-randomized model. No clear-cut recommendation was found in the literature for the difference between the average correlation coefficient (R_r_) of randomized models and the correlation coefficient (R) of non-randomized model. We have used a parameter R_p_^2^ [32] which penalizes the model R^2^ for the difference between squared mean correlation coefficient (R_r_^2^) of randomized models and squared correlation coefficient (R^2^) of the non-randomized model. 

In this paper, we demonstrate the usefulness of the parameters r_m_^2^ and R_p_^2^ in deriving predictive QSAR models. For this task, we have chosen three different data sets of moderate to large size and developed multiple models to indicate the suitability of the parameters in QSAR studies. It may be noted here that the purpose of this paper is not to develop new QSAR models for the data sets but to explore suitability of the novel parameters r_m_^2^ and R_p_^2^ in judging quality of predictive QSAR models.

## 2. Materials and Methods

### 2.1. The data sets and descriptors

In the present paper, three different data sets have been used for the QSAR model development: (1) CCR5 binding affinity data (IC_50_) of 119 piperidine derivatives [33,34,35,36]; (2) ovicidal activity data (LC_50_) of 90 2-(2′,6′-difluorophenyl)-4-phenyl-1,3-oxazoline derivatives [37] and (3) tetrahymena toxicity (IGC_50_) of 384 aromatic compounds [38]. For the three data sets (I, II and III), QSAR models were separately developed from genetic function approximation (GFA) technique [39] with 5,000 crossovers using Cerius2 version 4.10 software [40]. The descriptors used were from the classes of topological, structural, physicochemical and spatial types (*vide infra*). 

#### 2.1.1. Data set I

The CCR5 binding affinity data (IC_50_) of 119 piperidine derivatives [33,34,35,36] were converted to logarithmic scale [pIC_50_ = -logIC_50_ (mM)] and then used for the QSAR study. A total of 119 compounds were selected in our study, which are shown in Table 1. In cases of racemic compounds, only *S* configuration was considered for modeling because the *R* isomers are less potent [33,34]. For this data set, different classes of descriptors used were topological [Balaban index (Jx), kappa shape indices, Zagreb, Wiener, connectivity indices and E-state indices], structural [molecular weight (MW), numbers of rotatable bonds (Rotlbonds), number of hydrogen bond donors and acceptors and number of chiral centers], physicochemical [AlogP, AlogP98, LogP, MR and MolRef], spatial [RadOfGyration, Jurs, Shadow, Area, Density, Vm] and electronic [Apol, HOMO, LUMO and Sr] parameters. Definitions of all descriptors can be found at the Cerius2 tutorial available at the website http://www.accelrys.com.

#### 2.1.2. Data set II

The ovicidal activity data (LC_50_) of 90 2-(2′,6′-difluorophenyl)-4-phenyl-1,3-oxazoline derivatives [37] were converted to reciprocal logarithmic values [pLC_50_ = -logLC_50_ (M)] which were used for the QSAR analysis. There is only one region of structural variations in the compounds, which is the R position of the phenyl ring. Thus the present QSAR study explores the impact of substitutional variations at the 4-phenyl ring of the 1,3-oxazoline nucleus on the ovicidal activity of the compounds. The structures of the compounds and associated ovicidal activities are listed in Table 2. The range of the ovicidal activity values is quite wide (6.1 log units). For this data set, only topological descriptors (Balaban J, kappa shape, flexibility, subgraph count, connectivity, Wiener, Zagreb and E-sate) along with structural parameters [molecular weight (MW), numbers of rotatable bonds (Rotlbonds), number of hydrogen bond donors and acceptors and number of chiral centers] and hydrophobic substituent constant π were used for the model development.

#### 2.1.3. Data set III

Toxicity data (-log IGC_50_) (Table 3) determined against *T. pyriformis* [38] for 384 diverse compounds were used as the third data set. Different topological descriptors [ETA parameters [41,42] and non-ETA (Balaban J, kappa shape, flexibility, subgraph count, connectivity, Wiener, Zagreb, Hosoya and E-sate) parameters] were used to develop the models. 

### 2.2. Model development

A model’s predictive accuracy and confidence for different unknown chemicals varies according to how well the training set represents the unknown chemicals and how robust the model is in extrapolating beyond the chemistry space defined by the training set. So, the selection of the training set is significantly important in QSAR analysis. Predictive potential of a model on the new data set is influenced by the similarity of chemical nature between training set and test set [43]. The test set molecules will be predicted well when these molecules are very similar to the training set compounds. The reason is that the model has represented all features common to the training set molecules. In this paper, for the development of models for a particular data set, standardized descriptor matrix was subjected to cluster analysis by *K*-nearest neighbour method [44]. After clustering, test set compounds were selected from each cluster so that both test set and training set could represent all clusters and characteristics of the whole dataset. This approach (clustering) ensures that the similarity principle can be employed for the activity prediction of the test set. Based on clustering, each data set was divided into 50 combinations of training and test sets. In each case, 75% of the total compounds were selected as training set and remaining 25% were selected as test set. Models were developed from a training set using genetic function approximation and the best model was selected from the population of models obtained based on lack-of-fit score. The selected model was then validated internally by leave-one-out method and then externally by predicting the activity values of the corresponding test set. Based on the results obtained from multiple models which are derived based on different combinations of training and test sets, we have tried to evaluate performance of different validation parameters.

### 2.3. Statistical methods

#### 2.3.1. GFA

In this work, all models were developed using genetic function approximation (GFA) technique. Genetic algorithms are derived from an analogy with the evolution of DNA [39]. The genetic function approximation algorithm was initially anticipated by: 1) Holland’s genetic algorithm and 2) Friedman’s multivariate adaptive regression splines (MARS) algorithm. In this algorithm an individual or model is represented as one-dimensional string of bits. A distinctive feature of GFA is that it produces a population of models (e.g. 100), instead of generating a single model, as do most other statistical methods. Genetic algorithm makes superior models to those developed using stepwise regression techniques because it selects the basis functions genetically. Descriptors, which were selected by this algorithm, were subjected to multiple linear regression for generation of models. A “fitness function” or lack of fit (LOF) was used to estimate the quality of a model, so that best model receives the best fitness score. The error measurement term LOF is determined by the following equation:

(1) $$LOF=\frac{LSE}{{\left(1-\frac{c+d*p}{M}\right)}^{2}}$$

In Eq. (1), ‘c’ is the number of basis functions (other than constant term); ‘d’ is smoothing parameter (adjustable by the user); ‘M’ is number of samples in the training set; LSE is least squares error and ‘p’ is total numbers of features contained in all basis functions.

Once models in the population have been rated using the LOF score, the genetic cross over operation is repeatedly performed. Initially two good models are probabilistically selected as parents and each parent is randomly cut into two pieces and a new model (child) is generated using a piece from each parents. After many mating steps, i.e., genetic crossover type operation, average fitness of models in the population increases as good combinations of genes are discovered and spread through the population. It can build not only linear models but also higher-order polynomials, splines and Gaussians. In our present work, only linear terms have been used. For the development of genetic function approximation (GFA) model, Cerius2 version 4.10 [38] has been used. The mutation probabilities were kept at 5,000 iterations. Smoothness (*d*) was kept at 1.00. Initial equation length value was selected as 4 and the length of the final equation was not fixed. 

#### 2.3.2. Validation parameters

##### 2.3.2.1. Q^2^

In case of leave-one-out (LOO) cross-validation, each member of the sample in turn is removed, the full modeling method is applied to the remaining *n*-1 members, and the fitted model is applied to the holdback member. The LOO approach perturbs the data structure by removing 1/Nth compound in each crossvalidation round, thus, accomplishing an increasingly smaller perturbation with increasing N. Hence, the Q^2^ value of LOO approaches to that of R^2^, which is highly unsatisfactory [20].

Cross-validated squared correlation coefficient R^2^ (LOO-Q^2^) is calculated according to the formula:

(2) $$Q^{2}=1-\frac{\sum {\left(Y_{pred}-Y\right)}^{2}}{\sum {\left(Y-\bar{Y}\right)}^{2}}$$

In Eq. (2), Y_pred_ and Y indicate predicted and observed activity values respectively and $\bar{Y}$ indicate mean activity value. A model is considered acceptable when the value of Q^2^ exceeds 05.

##### 2.3.2.2. R^2^_pred_

Cross validation provides a reasonable approximation of ability with which the QSAR predicts the activity values of new compounds. However, external validation gives the ultimate proof of the true predictability of a model. In many cases, truly external data points being unavailable for prediction purpose, original data set compounds are divided into training and test sets [45], thus enabling external validation. This subdivision of the data set can be accomplished in many ways, but approximately similar ranges of the biological responses and structural properties and all available structural and/or physicochemical features should be represented in both training and test sets.

Equations are generated based on training set compounds and predictive capacity of the models is judged based on the predictive R^2^ (R^2^_pred_) values calculated according to the following equation:

(3) $$R^{2}{}_{pred}=1-\frac{\sum {\left(Y_{pred(test)}-Y_{(}{}_{test)}\right)}^{2}}{\sum {\left(Y_{(}{}_{test)}-{\bar{Y}}_{training}\right)}^{2}}$$

In Eq. (3), Y_pred(test)_ and Y_(test)_ indicate predicted and observed activity values respectively of the test set compounds and $\bar{Y}$_training_ indicates mean activity value of the training set. For a predictive QSAR model, the value of R^2^_pred_ should be more than 0.5.

##### 2.3.2.3. r_m_^2^

It has been previously shown [15] that R^2^_pred_ may not be sufficient to indicate external predictivity of a model. The value of R^2^_pred_ is mainly controlled by $\sum {\left(Y_{obs(test)}-{\bar{Y}}_{training}\right)}^{2}$, i.e., sum of squared differences between observed values of test set compounds and mean observed activity values of training data set. Thus, it may not truly reflect the predictive capability of the model on a new dataset. Besides this, a good value of squared correlation coefficient (r^2^) between observed and predicted values of the test set compounds does not necessarily mean that the predicted values are very near to corresponding observed activity (there may be considerable numerical difference between the values though maintaining an overall good intercorrelation). So, for better external predictive potential of the model, a modified r^2^ [r_m_^2^_(test)_] was introduced by the following equation [15]:

(4) $$r_{m(test)}^{2}=r^{2}*(1-\sqrt{r^{2}-r_{0}^{2}})$$

In Eq. (4), r_0_^2^ is squared correlation coefficient between the observed and predicted values of the test set compounds with intercept set to zero. The value of r^2^_m(test)_ should be greater than 0.5 for an acceptable model. 

Initially, the concept of r_m_^2^ was applied only to the test set prediction [15], but it can as well be applied for training set if one considers the correlation between observed and leave-one-out (LOO) predicted values of the training set compounds [39,40]. More interestingly, this can be used for the whole set considering LOO-predicted values for the training set and predicted values of the test set compounds. The r_m_^2^_(overall)_ statistic may be used for selection of the best predictive models from among comparable models. 

##### 2.3.2.4. R_p_^2^

Further statistical significance of the relationship between activity and the descriptors can be checked by randomization test (Y-randomization) of the models. This method is of two types: process randomization and model randomization. In case of process randomization, the values of the dependent variable are randomly scrambled and variable selection is done freshly from the whole descriptor matrix. In case of model randomization, the Y column entries are scrambled and new QSAR models are developed using same set of variables as present in the unrandomized model. For an acceptable QSAR model, the average correlation coefficient (R_r_) of randomized models should be less than the correlation coefficient (R) of non-randomized model. We have used a parameter R_p_^2^ [32] in the present paper, which penalizes the model R^2^ for the difference between squared mean correlation coefficient (R_r_^2^) of randomized models and squared correlation coefficient (R^2^) of the non-randomized model. The above mentioned novel parameter can be calculated by the following equation:

(5) $$R_{p}^{2}=R_{}^{2}*\sqrt{R_{}^{2}-R_{r}^{2}}$$

This novel parameter R_p_^2^ ensures that the models thus developed are not obtained by chance. We have assumed that the value of R_p_^2^ should be greater than 0.5 for an acceptable model. 

## 3. Results and Discussion

### 3.1. Data set I

The dataset (n = 119) was divided into training set of 89 compounds and test set of 30 compounds in 50 different combinations. Each of the 50 different training sets was then used for developing QSAR models using the genetic function approximation (GFA) technique. Each of the selected QSAR models was validated internally using the leave-one-out technique and externally using the corresponding test set compounds. All the models were also validated by the process randomization technique. From the internal validation technique, the value of Q^2^ was determined and from the external validation technique the value of R^2^_pred_ was calculated which were then used as the parameters for determining the model predictivity. Using the process randomization technique, the average of the correlation coefficients of the randomized models (R_r_) was compared with the correlation coefficient (R) of the non-randomized model. To penalize a model for the difference between the squared correlation coefficients of the randomized and the non-randomized models, the value R_p_^2^ was also calculated.

An illustration of the results obtained for each combination studied is given in Table 4. The Q^2^ values obtained for all the models are well above the stipulated value of 0.5 with model no. 39 showing the highest Q^2^ value of 0.701. However, external validation of the models showed a wide range of variation in the values of R^2^_pred_. A very low value of R^2^_pred_ is obtained for models showing high values of Q^2^ while models with moderate values of Q^2^ showed a similarly moderate values of R^2^_pred_. The value of R^2^_pred_ for model no. 39 is only 0.240 which is far below the stipulated acceptable value of 0.5 although the model gives the maximum value of Q^2^. Similarly model no. 12 gives the lowest value of R^2^_pred_ (0.117) in spite of having a quite acceptable value of Q^2^ (0.632). On the contrary, only model nos. 3, 6, 10, 11, 15, 18, 29, 37, 41 and 42 having Q^2^ values just exceeding 0.5 give values of R^2^_pred_ above 0.5. Again for model nos. 41 and 42, the value of R^2^_pred_ is greater the value of Q^2^. Thus it may be inferred that very a high value of Q^2^ does not indicate the model to be highly predictive while determining the activity of external dataset and also a model with high external predictivity may be poorly predictive internally. Thus the parameter, r_m_^2^_(overall)_, was used which penalizes a model for large differences in observed and predicted activity values of the congeners. A model may be considered satisfactory when r_m_^2^_(overall)_ is greater than 0.5.

**Figure 1:**
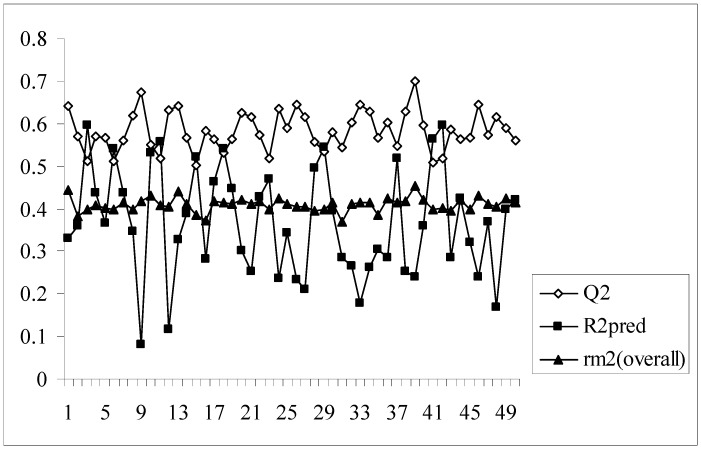
Comparative plots of Q^2^, R^2^_pred_ and r_m_^2^_(overall)_ values of 50 models (data set I).

As we know, high or acceptable values of the two parameters, Q^2^ and R^2^_pred_, may be obtained as long as a moderate overall correlation is maintained between the observed and predicted activity values even if there is a considerable difference between them. The parameter r_m_^2^_(overall)_ determines whether the predicted activities are really close to the observed values or not since high values of Q^2^ and R^2^_pred_ does not necessarily mean that the predicted values are very close to the observed ones. The value of r_m_^2^_(overall)_ is a good compromise between a high value of Q^2^ and a low value of R^2^_pred_ and *vice versa*. For models showing high acceptable values of Q^2^ but very low values of R^2^_pred_ (below 0.5) and *vice versa*, it becomes difficult to conclude whether the model is well predictive or not. Similarly, the results obtained here show that some of the models give high Q^2^ values while others give high R^2^_pred_ values. So, the selection of the best model becomes difficult. The value of r_m_^2^_(overall)_ takes into consideration predictions for both training and test set compounds and maintains a balance between the values of Q^2^ and R^2^_pred_. This fact can be well established from the Figure 1 showing a comparative plot of the values of Q^2^, R^2^_pred_ and r_m_^2^_(overall)_ for the 50 different models (trial nos. in x axis). The line showing the values of r_m_^2^_(overall)_ indicates that it can penalize a model with high Q^2^ but low R^2^_pred_. Furthermore, models with r_m_^2^_(overall)_ values greater than 0.5 may be considered acceptable. Thus, in this dataset, although some of the models are acceptable considering the values of the conventional parameters (Q^2^ and R^2^_pred_), none of the models satisfy the value of r_m_^2^_(overall)_. So none of the models obtained using the present descriptor matrix appears to be truly predictive. 

**Figure 2:**
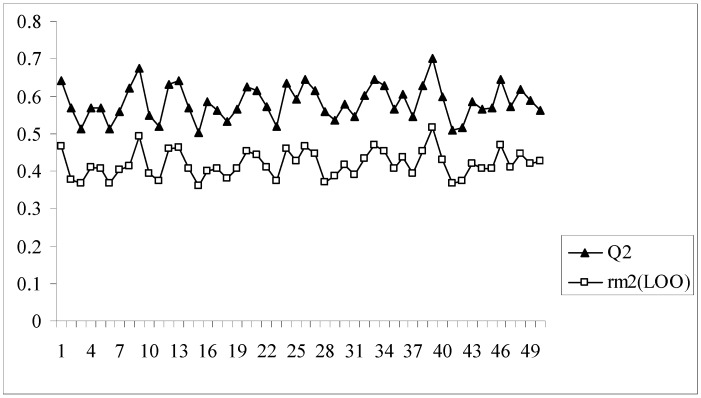
Comparative plots of Q^2^ and r_m_^2^_(LOO)_ values of 50 models (data set I).

In all the models developed for this dataset, there is a difference of at least 0.15 or more between the values of Q^2^ and r_m_^2^_(LOO)_, the latter parameter showing lower values. Model no. 8 having an acceptable value of Q^2^ (0.620) may appear to be quite good at a first glance, but this model bears the maximum difference between the values of Q^2^ and r_m_^2^_(LOO)_ (0.204). The r_m_^2^_(LOO)_ parameter for a given model indicates the extent of deviation of the LOO predicted activity values from the observed ones for the training set compounds. This implies that model 8, despite having an acceptable Q^2^, is not capable of accurately predicting the activities of some training set molecules (7 out of 89 training set compounds have LOO predicted residuals of more than 1 log unit) and this is reflected in the value of r_m_^2^_(LOO)_. Similar results are also obtained for model nos. 2, 9, 16, 28 and 39. Interestingly, model 39 has the maximum Q^2^ value (0.701) while the r_m_^2^_(LOO)_ value of this model is only 0.517. Figure 2 shows a comparative plot of the values of Q^2^ and r_m_^2^_(LOO)_ for the 50 different models.

The r_m_^2^_(test)_ parameter determines the extent of deviation of the predicted activity from the observed activity values of test set compounds where the predicted activity is calculated on the basis of the model developed using the corresponding training set. Model nos. 3, 6, 10, 11, 15, 18 and 41 show acceptable values of R^2^_pred_ and r_m_^2^_(test)_. 

**Figure 3:**
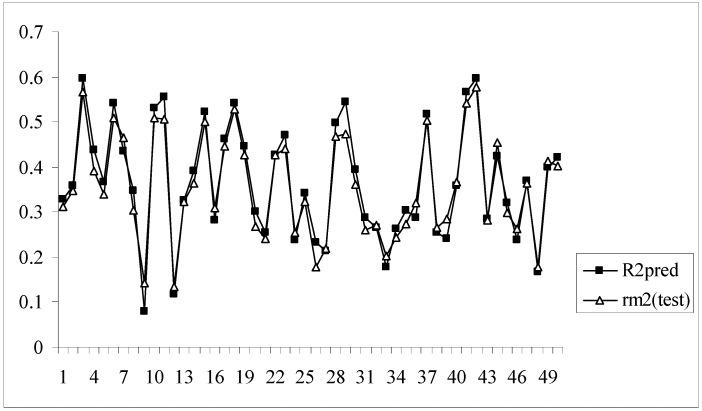
Comparative plots of R^2^_pred_ and r_m_^2^_(test)_ values of 50 models (data set I).

Moreover, for these models the difference between the value of R^2^_pred_ and r_m_^2^_(test)_ is very low (less than 0.1) indicating that the predicted activity values of the test set compounds obtained from the corresponding models are very close to the corresponding observed activities of the compounds. Figure 3 shows a comparative plot of the values of R^2^_pred_ and r_m_^2^_(test)_ for the 50 different models.

**Figure 4:**
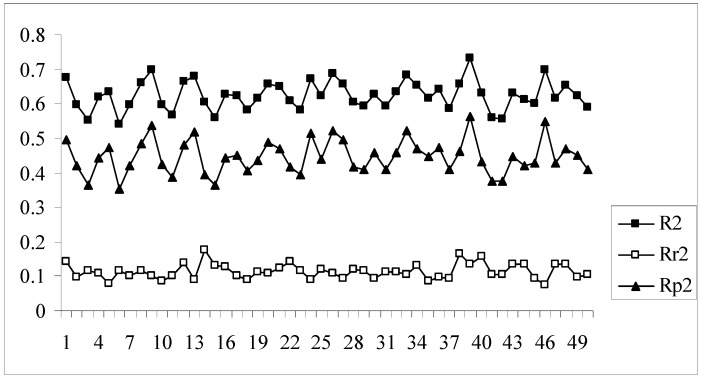
Comparative plots of R^2^, R_r_^2^ and R_p_^2^ values of 50 models (data set I).

The developed models were further validated by the process randomization technique. The values of R_r_^2^ and R^2^ were determined which were then used for calculating the value of R_p_^2^. Models with R_p_^2^ values greater than 0.5 are considered to be statistically robust. If the value of R_p_^2^ is less than 0.5, then it may be concluded that the outcome of the models is merely by chance and they are not at all well predictive for truly external datasets. Figure 4 shows a comparative plot of the values of R^2^, R_r_^2^ and R_p_^2^ for the 50 different models. In this work although some of the models satisfy the requirement for R_p_^2^, they do not achieve the stipulated value of r_m_^2^_(overall)_. Model nos. 9, 13, 24, 33, 39, 46 show acceptable values of R_p_^2^ (above 0.5) but at the same time none of them achieve the required value (0.5) of r_m_^2^_(overall)_. Thus it may be concluded that the different models obtained for this dataset using the given descriptor matrix do not appear to be truly predictive as none of them fulfills the requirements of both the parameters, r_m_^2^_(overall)_ and R_p_^2^, though many of them satisfy the conventional parameters, Q^2^ and R^2^_pred_. 

### 3.2. Data set II

The total data set (n=90) was divided into training set (n=68) and test (external evaluation) set (n=22) (75% and 25% respectively of the total number of compounds) in 50 different combinations, based on clusters obtained from *K*-means clustering applied on standardized topological, structural and physicochemical descriptor matrix. Models were generated with topological, structural and physicochemical descriptors of each of the training sets using GFA. The predictive potentials of those models were determined on the corresponding test sets. Each of the models were validated both internally (using Q^2^) and externally (using R^2^_pred_). The models were further validated using process randomization technique. A comparison of statistical quality parameters and validation parameters of the models are listed in Table 5. The Q^2^ values of model nos. 8, 37 and 42 did not cross the stipulated value, i.e., 0.5. But, the rest 47 models successfully crossed that threshold value. A very low value of R^2^_pred_ was obtained for models showing a high value of Q^2^ and *vice versa*, while models with a moderate value of Q^2^ showed a similarly moderate value of R^2^_pred_. As for example, model number 44 has the maximum leave-one-out (LOO) predicted variance (Q^2^ = 0.723), but the external predictive power of that model is very poor (R^2^_pred_ = 0.136), which is far less than the threshold value, i.e., 0.5. Similarly, model number 35 has also high internal predictive variance (Q^2^ = 0.704), but the external predictive potential of that model is very poor (R^2^_pred_ = -0.002). However, in case of model number 8, internal predictive variance (Q^2^ = 0.468) is quite less than the stipulated value, but the external predictive potential of that model (R^2^_pred_ = 0.714) is very good. However, the models with acceptable moderate values (greater than 0.5) of LOO predicted variance (Q^2^) like the model nos. 4, 6, 9, 13, 15, 17, 20, 22, 25, 28, 29, 34, 36, 46, 47, 50 showed satisfactory moderate values (higher than 0.5) of external predictive variance (R^2^_pred_). This dataset also implies that very high value of Q^2^ does not indicate the model to be highly predictive while determining the activity of external dataset and also a model with high external predictivity may be poorly predictive internally. Thus the values of r_m_^2^_(overall)_ were also calculated to penalize the models for large differences between observed and predictive values of the congeners.

**Figure 5:**
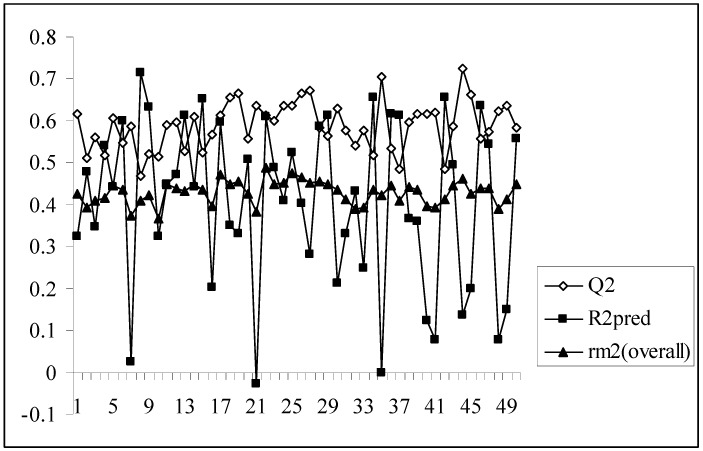
Comparative plots of Q^2^, R^2^_pred_ and r_m_^2^_(overall)_ values of 50 models (data set II).

Due to the wide distribution of the ovicidal activity among the congeners (range: 6.1 log units) acceptable values of the two parameters, Q^2^ and R^2^_pred_, were obtained in spite of bearing a considerable difference in numerical values of the observed and predicted activities. To penalize a model for large predicted residuals, r_m_^2^_(overall)_ was calculated. The results obtained here show that some of the models give high Q^2^ values while others give high R^2^_pred_ values, so for selecting the best model the values of r_m_^2^_(overall)_ were compared. The fact that the value of r^2^_m(overall)_ takes into consideration predictions for the whole dataset and maintains a compromise between the values of Q^2^ and R^2^_pred_ is established from the Figure 5 showing a comparative plot of the values of Q^2^, R^2^_pred_ and r_m_^2^_(overall)_ for the 50 different models. The line showing the values of r_m_^2^_(overall)_ indicates that it penalizes a model for large difference between Q^2^ and R^2^_pred_ values. Models with r_m_^2^_(overall)_ values greater than (or, at least near to) 0.5 may be considered acceptable. Thus, in this dataset, although some of the models are acceptable considering the values of the conventional parameters (Q^2^ and R^2^_pred_), yet none of the models satisfy the value of r^2^_m(overall)_. But, the value of r_m_^2^_(overall)_ of the model no. 22 (0.488) is very close to the predetermined criterion. 

**Figure 6:**
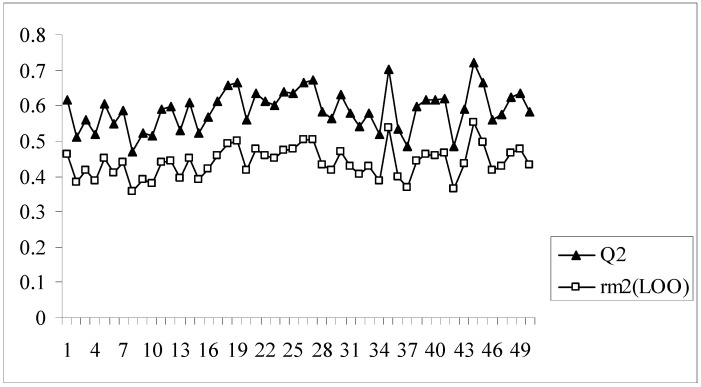
Comparative plots of Q^2^ and r_m_^2^_(LOO)_ values of 50 models (data set II).

**Figure 7:**
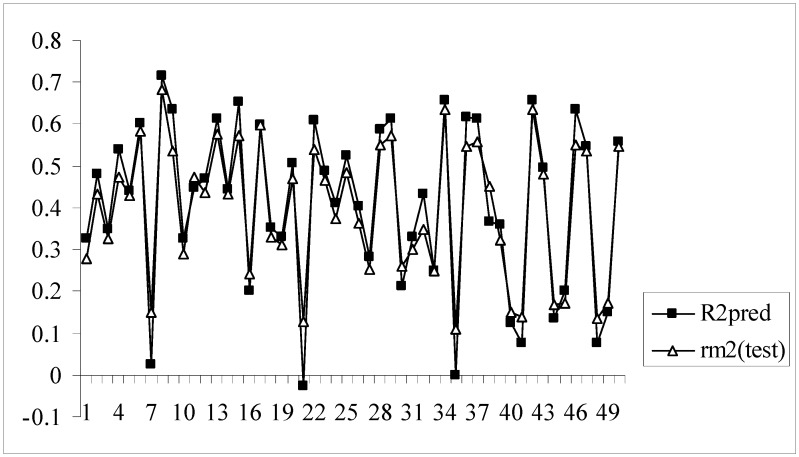
Comparative plots of R^2^_pred_ and r_m_^2^_(test)_ values of 50 models (data set II).

The r_m_^2^_(LOO)_ parameter for a given model is a measure of the extent of deviation of the LOO predicted activity values from the observed ones for the training set compounds. In all the models developed for this dataset, there is a difference of at least 0.111 or more between the values of Q^2^ and r_m_^2^_(LOO)_ and value of the latter parameter is always lower than the former. A very high value of Q^2^ may indicate the model to be well predictive internally but at the same time low value of r_m_^2^_(LOO)_ (below 0.5) for that model indicates that there exists a considerable difference between the observed and LOO predicted activity values. Hence, it may be considered that a model predictivity improves as the difference between these two parameters [Q^2^ and r_m_^2^_(LOO)_] reduces. Model number 44 has a considerably high value of Q^2^ (0.723) and thus the predictive potential of the model may appear to be a highly acceptable but the LOO predicted residuals of 13 compounds (out of 68) in the training set are more than 1 log unit. This has not been reflected in the Q^2^ value while r_m_^2^_(LOO)_ value of the model is comparatively much lower (0.551). Thus the parameter r_m_^2^_(LOO)_ has been able to capture the information on deviation of LOO predicted values from the observed ones for the training set compounds more efficiently and it may serve as a more strict parameter than Q^2^ for internal validation. Figure 6 shows a comparative plot of the values of Q^2^ and r_m_^2^_(LOO)_ for the 50 different models. Similarly, r_m_^2^_(test)_ parameter determines the extent of deviation of the predicted activity from the observed activity values for the test set compounds. Model number 25 has an acceptable value of R^2^_pred_ (0.525) but the predicted residuals of 6 compounds (out of 22 compounds) in the test set are more than 1 log unit. Though the model bears an acceptable value of R^2^_pred_ (0.525), the model can not be concluded to be truly predictive externally and it has not been reflected in the value of R^2^_pred_. However, the value of r_m_^2^_(test)_ (0.484) has not crossed the threshold value of 0.5. Thus r_m_^2^_(test)_ appears to be a more stringent parameter than R^2^_pred_ for external validation. Figure 7 shows a comparative plot of the values of R^2^_pred_ and r_m_^2^_(test)_ for the 50 different models.

**Figure 8:**
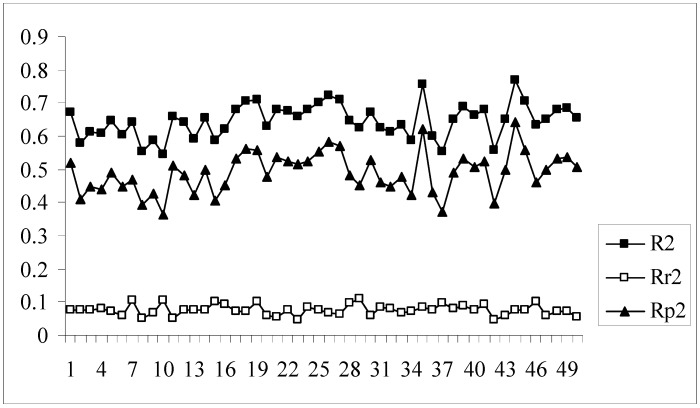
Comparative plots of R^2^, R_r_^2^ and R_p_^2^ values of 50 models (data set II).

Robustness of the models relating the ovicidal activity with selected descriptors was judged by randomization (Y-randomization) of the model development process. To penalize the model R^2^ for the difference between R_r_^2^ and R^2^, R_p_^2^ was also determined. Figure 8 shows a comparative plot of the values of R^2^ and R_p_^2^ for the 50 different models. In this data set, the values of R_p_^2^ of 23 models out of 50 models crossed the threshold value of 0.5 and thus those models may be considered to be statistically robust. But, at the same time if the value of r_m_^2^_(overall)_ is considered then those models are not acceptable since none of them achieve the required value (0.5) of r_m_^2^_(overall)_. But, we mentioned previously that the value of r_m_^2^_(overall)_ of the model number 22 (0.488) is very close to the required value (0.5) and that model has also acceptable value of R_p_^2^ (0.522). These results thus suggest that this combination of training and test sets is the best one out of the 50 combinations.

### 3.3. Data set III

Based on cluster analysis applied on standardized descriptor matrix, the dataset (n=384) was divided into training set of 288 compounds and test set of 96 compounds in 50 different combinations. Each of the 50 different training sets was then used for developing QSAR models using the genetic function approximation (GFA) technique. Each of the best QSAR models obtained from training set was validated internally using the leave-one-out technique and externally using the corresponding test set compounds to determine the values of Q^2^ and R^2^_pred_ respectively which were used for determining model predictivity. The models were also validated by the process randomization technique and the values of R_r_ and R were calculated to obtain the value of R_p_^2^ which penalizes the models for differences in the values of R_r_^2^ and R^2^. 

The results of the above-mentioned 50 different trials are shown in Table 6. For this dataset all the 50 models passed the critical value (0.5) for Q^2^ (Q^2^ ranging from 0.660 to 0.774) while only two models (37, 23) failed to cross the 0.5 limit for R^2^_pred_ (R^2^_pred_ ranging from 0.384 to 0.834). For all the models the difference between R^2^ and Q^2^ values is not very high (less than 0.3). As illustrated in Table 6 that models with maximum internal predictive variance do not correspond to model with maximum external prediction power and vice versa. Trial 50 has the highest Q^2^ value (0.774) but the corresponding predictive R^2^ value is 0.596. On the other hand trial 45 shows the maximum value of R^2^_pred_ (0.834) and the corresponding Q^2^ value is 0.677. Models with small differences in the above two parameters values are observed in the trials (6, 10, 13, 18, 27, 33, 35, 37 and 40). Large differences in the values of the parameters are observed in trials 1, 9, 15, 20, 25, 42 and 50. Except models 37 and 23 all the other models are statistically acceptable (Q^2^> 0.5 and R^2^_pred_> 0.5). Thus for selecting the best model, values of r_m_^2^_(overall)_ for all the models was determined. As shown above, this parameter penalizes a model for large differences in observed and predicted activity values of the congeners. 

**Figure 9:**
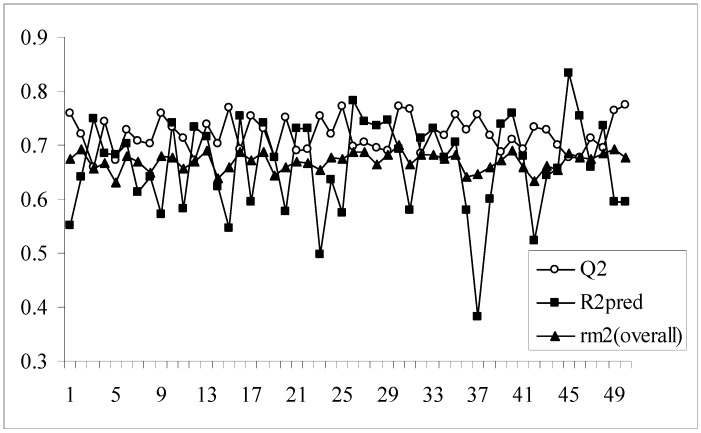
Comparative plots of Q^2^, R^2^_pred_ and r_m_^2^_(overall)_ values of 50 models (data set III).

Similar to the results obtained for the two datasets mentioned above, Table 6 also corresponds to the fact that the parameter, r_m_^2^_(overall)_ penalizes a model for wide difference in the values of Q^2^ and R^2^_pred_. This fact can be further established from the Figure 9 showing a comparative plot of the values of Q^2^, R^2^_pred_ and r_m_^2^_(overall)_ for the 50 different models. For this data set all the models have the r_m_^2^_(overall)_ value above 0.5 (0.631-0.699). The best model according to r^2^_m(overall)_ is obtained from trial 30 and the corresponding Q^2^ and R^2^_pred_ values are 0.771 and 0.692 respectively. It is obvious none of the parameter (Q^2^ and R^2^_pred_ ) has its maximum value for this trial, however the overall parameter, r_m_^2^_(overall)_, shows a maximum.

**Figure 10:**
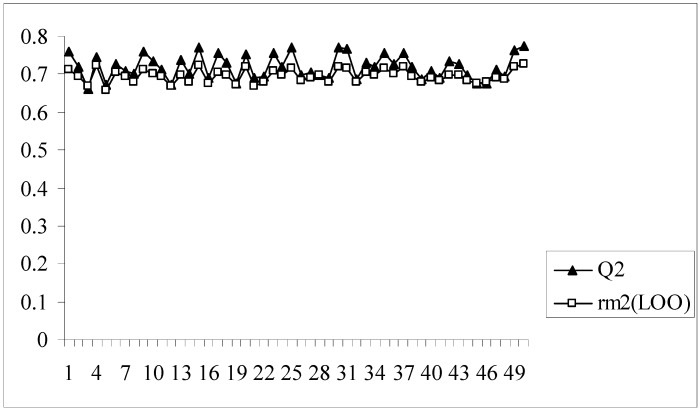
Comparative plots of Q^2^ and r_m_^2^_(LOO)_ values of 50 models (data set III).

**Figure 11:**
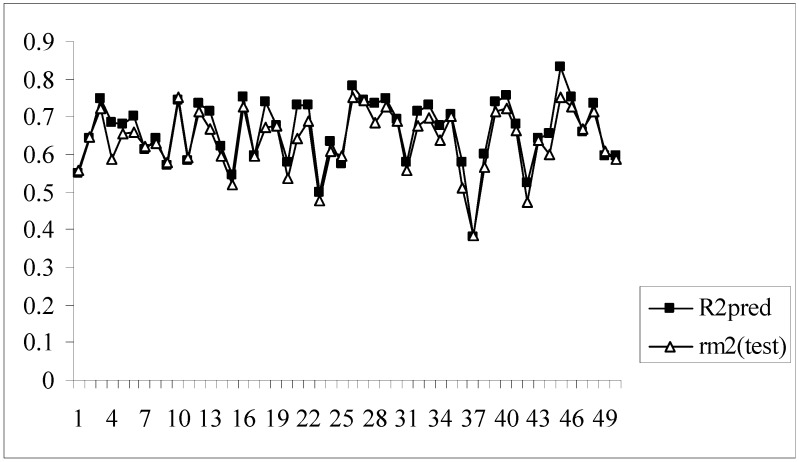
Comparative plots of R^2^_pred_ and r_m_^2^_(test)_ values of 50 models (data set III).

Besides r_m_^2^_(overall),_ we have calculated r_m_^2^_(test)_ and r_m_^2^_(LOO)_ values for all the 50 trials. These two parameters signify the differences between the observed and predicted activities of the test and training set compounds in that order. For an ideal predictive model, the difference between R^2^_pred_ and r_m_^2^_(test)_ and difference between Q^2^ and r_m_^2^_(LOO)_ should be low. Large difference between the values of R^2^_pred_ and r_m_^2^_(test)_ and that between Q^2^ and r_m_^2^_(LOO)_ will ultimately lead to poor values of r_m_^2^_(overall)_ parameter. Figure 10 shows a comparative plot of the values of Q^2^ and r_m_^2^_(LOO)_ for the 50 different models while Figure 11 shows a comparative plot of the values of R^2^_pred_ and r_m_^2^_(test)_ for the 50 different models. For this data set, the difference between Q^2^ and r_m_^2^_(LOO)_ is quite less (-0.008 to 0.057) and that between R^2^_pred_ and r_m_^2^_(test)_ is also very less (-0.019 to 0.099). Thus indicates that the models obtained for this data set using the topological descriptors are quite robust and predictive.

**Figure 12:**
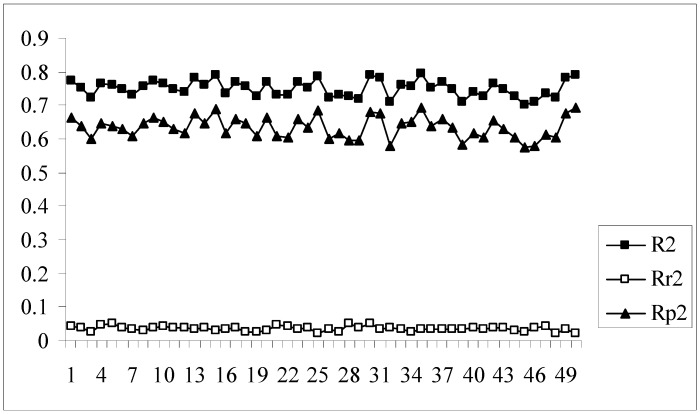
Comparative plots of R^2^, R_r_^2^ and R_p_^2^ values of 50 models (data set III).

Further validation of the developed models by the randomization technique and the subsequent calculation of the value of R_p_^2^ yielded results showing that none of the models developed were by chance only and the models were statistically robust. Figure 12 shows a comparative plot of the values of R^2^ and R_p_^2^ for the 50 different models. In this dataset, values of R_p_^2^ for all the models are well above the stipulated value of 0.5 (R_p_^2^: 0.574-0.695) as shown in Table 6. Moreover since all the models showed acceptable values of r^2^_m(overall)_, it can be concluded that besides being robust all the models developed are well predictive.

### 3.4. Overview

The QSAR models obtained for all the datasets considered in this work and their subsequent validation show that the parameters which are traditionally calculated during internal and external validation of models (Q^2^ and R^2^_pred_) are not enough for determining whether the model obtained is acceptable or not from the view point of predictability. Thus, additional parameters are needed for selecting the best model and confirming that the model obtained is robust and not by mere chance. These criteria are fulfilled by the parameters r^2^_m(overall)_ and R_p_^2^. The value of r^2^_m(overall)_ determines whether the range of predicted activity values for the whole dataset of molecules are really close to the observed activity or not. Since the value of r^2^_m(overall)_ takes into consideration the whole dataset, it penalizes models for differences between the values of Q^2^ and R^2^_pred_ enabling one to select the best predictive model. The value of R_p_^2^, on the contrary, determines whether the model obtained is really robust or obtained as a result of chance only. Hence it can be inferred that if the values of r_m_^2^_(overall)_ and R_p_^2^ are equal to or above 0.5 (or at least near 0.5), a QSAR model can be considered acceptable. Finally it can be inferred that selection of QSAR models on the basis of Q^2^ and R^2^_pred_ may mislead the search for the ideally predictive model. The selection of robust and well predictive QSAR models may be done merely on the basis of the two parameters, r_m_^2^_(overall)_ and R_p_^2^, in addition to the conventional parameters. Consideration of these parameters helps one to develop more stringent models which can be successfully applied to predict the activities of molecules in a truly external dataset.

**Figure 13:**
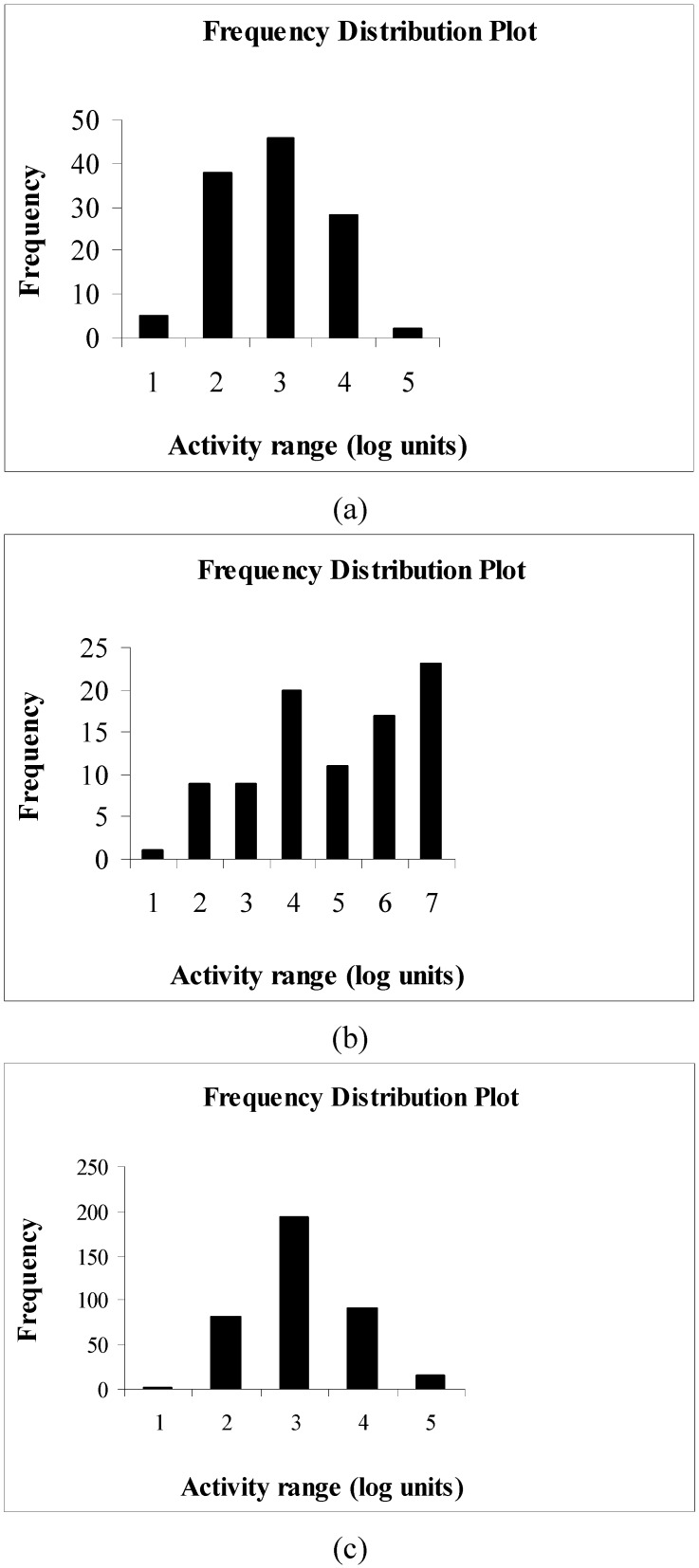
Frequency distribution of compounds for different relative ranges of biological activity data (from low to high in log units): (a) data set I, (b) data set II, (c) data set III.

The results obtained from the present study on the three data sets show that only the third data set gives Q^2^ values very close to corresponding r_m_^2^_(LOO)_ values (Figure 10) while other two data sets show large fluctuations of Q^2^ values from the corresponding r_m_^2^_(LOO)_ values, the latter being always less than the former (Figure 2 and Figure 6). The reason may be the quality of the biological activity data, apart from the performance of the selected descriptors to explain a particular biological activity in relation to the structural features. In case of data sets I and III, the biological activity data are satisfactorily distributed (Figure 13), while in case of data set II the distribution is not satisfactory. Thus, for data set I, the differences between Q^2^ and corresponding r_m_^2^_(LOO)_ values may be attributed to the inability of the selected descriptors to satisfactorily explain the change of biological activity values with changes in structural features while in case of the second data set, it may be due to unsatisfactory distribution of the biological activity values. 

It may be noted here that r_m_^2^ values do not take into account the number of predictor variables included in a model. When different models, having different number of predictor variables are compared then it may be very difficult to determine which one is the best model as r_m_^2^ does not consider the number of predictor variables used. To solve this problem, another parameter [r_m_^2^_(overall)_(adjusted)] may be calculated in a manner similar to the adjusted R^2^ (R^2^_a_):

(6) $$r_{m(overall)}^{2}(adjusted)=\frac{(n-1)*r_{m(overall)}^{2}-p}{n-p-1}$$

In Eq. (6), n is the total number of compounds and p is the number of predictor variables. The values of the parameter r_m_^2^_(overall)_(adjusted) for all the models of data sets I, II and III have been shown in Table 4, Table 5 and Table 6 respectively.

## 4. Conclusions

QSAR models have been traditionally tested for their predictive potential using internal (Q^2^) and external validation (R^2^_pred_) parameters. The present study shows that even in presence of considerable differences between observed and LOO predicted values of the training set compounds, Q^2^ value may be considerably high thus not reflecting bad predictions for some compounds. The parameter r_m_^2^_(LOO)_ is a stricter metric for internal validation than Q^2^. Similarly r_m_^2^_(test)_ appears to be a better metric to denote external predictivity than the traditional parameter R^2^_pred_. The parameter r_m_^2^_(overall)_ is unique in that it considers predictions for both training and test set compounds and its value is not obtained from prediction of limited number of test set compounds as is the case for R^2^_pred_. In addition to this, r_m_^2^_(overall)_ helps to identify the best model from among comparable models, especially when different models show different patterns in internal and external predictivity. The parameter R_p_^2^ penalizes model R^2^ for large differences between determination coefficient of nonrandom model and square of mean correlation coefficient of random models in case of a randomization test and thus confirms whether a model has been obtained by chance or not. A model can be considered robust, truly predictive and not obtained by chance when the parameters r_m_^2^ (all three variants) and R_p_^2^ cross the minimum limit of 0.5 (or at least near 0.5). Thus, in addition to the traditional validation parameters, tests for r_m_^2^ and R_p_^2^ should be carried out for a more stringent test of validation of predictive QSAR models, especially when a regulatory decision is involved.

## Figures and Tables

**Table 1:**
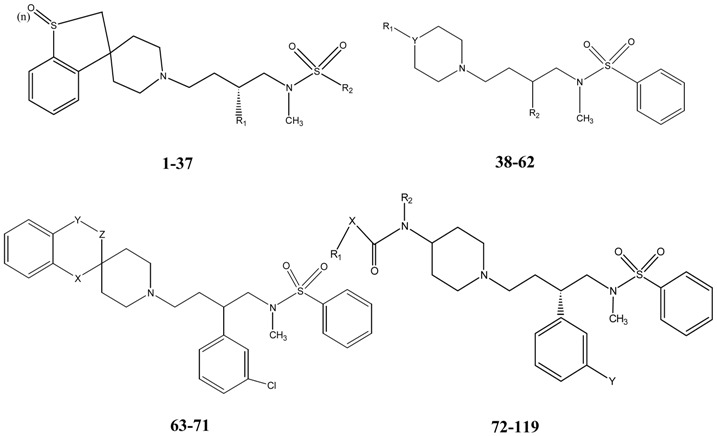
Structural features and CCR5 binding affinities of piperidine containing compounds.

Sl. No.		Structural Features					CCR5 binding affinity (-logIC_50_(mM))
	Number of oxygen atoms (n)	R1	R2	Y	X	Y-Z	Observed [33,34,35,36]
1	0	(S)-3,4-Cl_2_-phenyl	Phenyl	-	-	-	3.000
2	1	(S)-3,4-Cl_2_-phenyl	Phenyl	-	-	-	4.456
3	2	(S)-3,4-Cl_2_-phenyl	Phenyl	-	-	-	4.000
4	1	(S)-3,4-Cl_2_-phenyl	2-Thienyl	-	-	-	4.222
5	2	(S)-3,4-Cl_2_-phenyl	2-Thienyl	-	-	-	3.921
6	1	(S)-3,4-Cl_2_-phenyl	Dimethylamino	-	-	-	3.469
7	1	(S)-3,4-Cl_2_-phenyl	Benzyl	-	-	-	3.229
8	1	(S)-3,4-Cl_2_-phenyl	Methyl	-	-	-	3.071
9	1	(S)-3,4-Cl_2_-phenyl	n-Octyl	-	-	-	2.854
10	1	(S)-3,4-Cl_2_-phenyl	Cyclopentyl	-	-	-	4.000
11	1	(S)-3,4-Cl_2_-phenyl	Cyclohexyl	-	-	-	4.000
12	1	(S)-3,4-Cl_2_-phenyl	2-Cl-phenyl	-	-	-	4.097
13	1	(S)-3,4-Cl_2_-phenyl	3-Cl-phenyl	-	-	-	4.155
14	1	(S)-3,4-Cl_2_-phenyl	4-Cl-phenyl	-	-	-	4.398
15	2	(S)-3,4-Cl_2_-phenyl	3-NO_2_-phenyl	-	-	-	3.824
16	2	(S)-3,4-Cl_2_-phenyl	4-NO_2_-phenyl	-	-	-	4.222
17	1	(S)-3,4-Cl_2_-phenyl	4-MeO-phenyl	-	-	-	4.398
18	1	(S)-3,4-Cl_2_-phenyl	4-Phenyl-phenyl	-	-	-	4.398
19	1	(S)-3,4-Cl_2_-phenyl	Naphth-1-yl	-	-	-	3.444
20	1	(S)-3,4-Cl_2_-phenyl	Naphth-2-yl	-	-	-	4.222
21	1	(S)-3,4-Cl_2_-phenyl	Indan-5-yl	-	-	-	4.155
22	1	(S)-3,4-Cl_2_-phenyl	Pyridin-3-yl	-	-	-	4.000
23	1	(S)-3,4-Cl_2_-phenyl	Quinolin-8-yl	-	-	-	4.046
24	1	(S)-3,4-Cl_2_-phenyl	Quinolin-3-yl	-	-	-	3.921
25	1	(S)-3,4-Cl_2_-phenyl	1-Me-imidazol-4-yl	-	-	-	3.469
26	0	(R/S)-phenyl	Phenyl	-	-	-	3.347
27	1	(R/S)-phenyl	Phenyl	-	-	-	4.456
28	2	(R/S)-phenyl	Phenyl	-	-	-	4.523
29	1	(R/S)-2-Cl-phenyl	Phenyl	-	-	-	2.699
30	2	(R/S)-2-Cl-phenyl	Phenyl	-	-	-	2.886
31	0	(S)-3-Cl-phenyl	Phenyl	-	-	-	3.569
32	1	(S)-3-Cl-phenyl	Phenyl	-	-	-	5.000
33	2	(S)-3-Cl-phenyl	Phenyl	-	-	-	4.824
34	1	(S)-4-Cl-phenyl	Phenyl	-	-	-	3.569
35	1	(S)-4-F-phenyl	Phenyl	-	-	-	3.244
36	1	(R/S)-3,5- Cl_2_-phenyl	Phenyl	-	-	-	4.046
37	2	(R/S)-3,5- Cl_2_-phenyl	Phenyl	-	-	-	3.959
38	-	Phenyl	(R/S)-Phenyl	-CH-	-	-	3.921
39	-	Phenyl	(R/S)-2-Cl-phenyl	-CH-	-	-	2.523
40	-	Phenyl	(S)-3-Cl-phenyl	-CH-	-	-	4.523
41	-	Phenyl	(S)-4-F-phenyl	-CH-	-	-	3.000
42	-	Phenyl	(R/S)-3,5-Cl_2_-phenyl	-CH-	-	-	3.523
43	-	Phenyl	(R/S)-3-F-phenyl	-CH-	-	-	4.000
44	-	Phenyl	(R/S)-3-Me-phenyl	-CH-	-	-	4.097
45	-	Phenyl	(R/S)-3-Et-phenyl	-CH-	-	-	3.959
46	-	Phenyl	(R/S)-3-CF_3_-phenyl	-CH-	-	-	3.301
47	-	Phenyl	(R/S)-4-Me-phenyl	-CH-	-	-	3.699
48	-	Phenyl	(R/S)-3,5-Me_2_-phenyl	-CH-	-	-	3.796
49	-	Phenyl	(R/S)-3,4-F_2_-phenyl	-CH-	-	-	3.244
50	-	Phenyl	(R/S)-3,4-Me_2_-phenyl	-CH-	-	-	4.222
51	-	Phenyl	(R/S)-3-Me-4-F-phenyl	-CH-	-	-	3.745
52	-	Phenyl	(R/S)-3-F-4-Me-phenyl	-CH-	-	-	3.959
53	-	Phenyl	3-Cl-phenyl	-N-	-	-	3.155
54	-	2-Methyl-phenyl	3-Cl-phenyl	-N-	-	-	2.620
55	-	2-Methyl-phenyl	3-Cl-phenyl	-CH-	-	-	3.398
56	-	2-MeO-phenyl	3-Cl-phenyl	-CH-	-	-	4.155
57	-	3-CF_3_-phenyl	3-Cl-phenyl	-CH-	-	-	3.921
58	-	4-Cl-phenyl	3-Cl-phenyl	-CH-	-	-	3.699
59	-	4-F-phenyl	3-Cl-phenyl	-CH-	-	-	4.602
60	-	Benzyl	3-Cl-phenyl	-CH-	-	-	3.602
61	-	C_6_H_5_CH_2_CH_2_	3-Cl-phenyl	-CH-	-	-	4.187
62	-	C_6_H_5_CH_2_CH_2_CH_2_	3-Cl-phenyl	-CH-	-	-	5.301
63	-	-	-	-	-^a^	-CH_2_CH_2_-	3.745
64	-	-	-	-	-^a^	-NHCH_2_-	4.301
65	-	-	-	-	-^a^	-C(O)CH_2_-	5.301
66	-	-	-	-	-^a^	-C(O)NH-	4.347
67	-	-	-	-	-^a^	-C(O)N(Me)	4.000
68	-	-	-	-	-^a^	-C(O)NHCH_2_-	4.456
69	-	-	-	-	-^a^	-NHC(O)CH_2_-	4.456
70	-	-	-	-	-^a^	-CH(OH)CH_2_-	4.000
71	-	-	-	-	-CH_2_-	-O-	3.585
72	-	Me	H	H	O	-	3.000
73	-	t-Bu	H	H	O	-	3.000
74	-	t-Bu	Et	H	O	-	4.523
75	-	Me	Me	H	O	-	3.824
76	-	Me	Et	H	O	-	4.398
77	-	Me	n-Pr	H	O	-	4.699
78	-	Me	n-Bu	H	O	-	4.824
79	-	Me	n-C_6_H_13_	H	O	-	5.000
80	-	Me	c-C_6_H_11_-CH_2_	H	O	-	5.222
81	-	Me	Bn	H	O	-	4.000
82	-	Et	c-C_6_H_11_-CH_2_	H	O	-	4.456
83	-	Bn	c-C_6_H_11_-CH_2_	H	O	-	3.097
84	-	Et	Et	H	O	-	4.398
85	-	t-Bu	Et	H	O	-	4.602
86	-	c-C_6_H_11_-CH_2_	Et	H	O	-	4.824
87	-	Ph	Et	H	O	-	5.000
88	-	Bn	Et	H	O	-	5.699
89	-	Bn	Et	Cl	O	-	5.699
90	-	Bn	Me	H	O	-	5.301
91	-	Bn	n-Pr	H	O	-	5.699
92	-	Bn	n-Pr	Cl	O	-	5.398
93	-	Bn	n-Bu	H	O	-	5.301
94	-	Bn	Allyl	H	O	-	5.824
95	-	2-Me-C_6_H_4_-CH_2_	n-Pr	H	O	-	5.398
96	-	3-Me-C_6_H_4_-CH_2_	n-Pr	H	O	-	5.523
97	-	4-Me-C_6_H_4_-CH_2_	n-Pr	H	O	-	5.523
98	-	4-CF_3_-C_6_H_4_-CH_2_	n-Pr	H	O	-	5.222
99	-	4-NO_2_-C_6_H_4_-CH_2_	n-Pr	H	O	-	5.824
100	-	4-NO_2_-C_6_H_4_-CH_2_	Allyl	H	O	-	5.699
101	-	4-NO_2_-C_6_H_4_-CH_2_	Allyl	Cl	O	-	5.699
102	-	3-NH_2_COC_6_H_4_-CH_2_	n-Pr	H	O	-	6.097
103	-	4-NH_2_COC_6_H_4_-CH_2_	n-Pr	H	O	-	5.699
104	-	4-NH_2_COC_6_H_4_-CH_2_	n-Pr	Cl	O	-	5.523
105	-	Bn	n-Pr	H	O	-	5.699
106	-	Me	H	H	NH	-	3.000
107	-	Me	Et	H	NH	-	3.921
108	-	Bn	H	H	NH	-	4.000
109	-	Bn	n-Pr	H	NH	-	5.602
110	-	Ph	n-Pr	H	NH	-	5.398
111	-	Bn	n-Pr	H	N-Me	-	4.699
112	-	(S)-α-Me-Bn	n-Pr	H	NH	-	4.125
113	-	4-NO_2_-Bn	Allyl	H	NH	-	6.125
114	-	Me	Et	H	-	-	3.921
115	-	Ph	n-Pr	H	-	-	4.000
116	-	Bn	n-Pr	H	-	-	5.523
117	-	PhOCH_2_	n-Pr	H	-	-	5.398
118	-	PhCH_2_CH_2_	n-Pr	H	-	-	4.699
119	-	4-NO_2_-Bn	Allyl	H	-	-	5.699

^a^The X feature in these structures is a single bond.

**Table 2:**
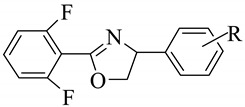
Structural features and ovicidal activity of 2-(2′,6′-difluorophenyl)-4-phenyl-1,3-oxazoline derivatives.

Sl. No.	Substitution (R)	Ovicidal activity
		Observed [37]
1	H	4.71
2	2-CH_3_	3.74
3	2-Et	4.76
4	2-OCH_3_	3.76
5	2-OEt	3.78
6	2-F	4.74
7	2-Cl	5.77
8	3-CH_3_	3.74
9	3-Et	3.76
10	3-OCH_3_	4.76
11	3-OEt	4.78
12	3-F	4.74
13	3-Cl	4.77
14	4-CH_3_	5.74
15	4-Et	7.76
16	4-i-Pr	7.78
17	4-n-Bu	8.8
18	4-i-Bu	8.8
19	4-t-Bu	8.8
20	4-n-C_6_H_13_	8.84
21	4-n-C_8_H_17_	8.87
22	4-n-C_10_H_21_	8.9
23	4-n-C_12_H_25_	8.93
24	4-n-C_15_H_31_	7.97
25	4-OH	3.74
26	4-OCH_3_	4.76
27	4-OEt	7.78
28	4-O-iPr	7.8
29	4-n-Bu	8.82
30	4-O-n-C_8_H_17_	8.89
31	4-O-n-C_10_H_21_	8.92
32	4-O-n-C_13_H_27_	7.96
33	4-O-n-C_14_H_29_	6.97
34	4-OCF_3_	7.84
35	4-OCH_2_CF_3_	8.85
36	4-SCH_3_	5.79
37	4-S-i-Pr	5.82
38	4-S-NC_9_H_19_	6.92
39	4-S(=O)CH_3_	3.81
40	4-SO_2_CH_3_	2.83
41	4-F	5.74
42	4-Cl	7.77
43	4-Br	7.83
44	4-CF_3_	6.82
45	4-N(CH_3_)_2_	3.78
46	4-Si(CH_3_)_3_	8.82
47	2-CH_3_, 4-CH_3_	3.76
48	2-CH_3_, 4-n-C_8_H_17_	8.89
49	2-CH_3_, 4-Cl	5.79
50	2-OCH_3_, 4-t-Bu	7.84
51	2-OCH_3_, 4-n-C_8_H_17_	6.9
52	2-OCH_3_, 4-n-C_9_H_19_	7.92
53	2-OCH_3_, 4-n-C_10_H_21_	6.93
54	2-OCH_3_, 4-F	5.79
55	2-OCH_3_, 4-Cl	5.81
56	2-OEt, 4-i-Pr	6.84
57	2-OEt, 4-t-Bu	7.86
58	2-OEt, 4-n-C_5_H_11_	8.87
59	2-OEt, 4-F	7.81
60	2-OEt, 4-Cl	5.83
61	2-OEt, 4-Br	5.88
62	2-O-n-Pr, 4-i-Pr	8.86
63	2-O-n-Pr, 4-t-Bu	7.87
64	2-O-n-Pr, 4-n-C_5_H_11_	7.89
65	2-O-n-Bu, 4-t-Bu	6.89
66	2-O-n-Bu, 4-F	8.84
67	2-O-n-Hex, 4-t-Bu	5.92
68	2-F, 4-Et	5.79
69	2-F, 4-n-C_6_H_13_	8.86
70	2-F, 4-n-C_7_H_15_	8.88
71	2-F, 4-n-C_8_H_17_	8.89
72	2-F, 4-n-C_10_H_21_	7.92
73	2-F, 4-n-C_12_H_25_	6.95
74	2-F, 4-F	6.77
75	2-F, 4-Cl	8.79
76	2-Cl, 4-Et	7.81
77	2-Cl, 4-i-Bu	8.84
78	2-Cl, 4-n-C_6_H_13_	8.88
79	2-Cl, 4-n-C_8_H_17_	8.91
80	2-Cl, 4-n-C_10_H_21_	5.94
81	2-Cl, 4-n-C_12_H_25_	5.97
82	2-Cl, 4-F	5.79
83	2-Cl, 4-Cl	6.82
84	3-CH_3_, 4-CH_3_	4.76
85	3-F, 4-n-C_6_H_13_	5.86
86	3-F, 4-F	5.77
87	3-F, 4-Cl	6.79
88	3-Cl, 4-n-C_6_H_13_	5.88
89	3-Cl, 4-F	5.79
90	3-Cl, 4-Cl	5.82

**Table 3:** Toxicity (-log IGC_50_) of diverse compounds against *T. Pyriformis.*

Sl. No	Name	Toxicity [38]
1	3-Aminobenzyl alcohol	-1.13
2	2-Aminobenzyl alcohol	-1.07
3	Benzyl alcohol	-0.83
4	4-Hydroxyphenethyl alcohol	-0.83
5	4-Aminobenzyl cyanide	-0.76
6	2-Nitrobenzamide	-0.72
7	4-Hydroxy-3-methoxybenzyl alcohol	-0.7
8	2-Methoxyaniline	-0.69
9	(*sec*)-Phenethyl alcohol	-0.66
10	1,3-Dihydroxybenzene	-0.65
11	1-Phenyl-2-propanol	-0.62
12	Phenethyl alcohol	-0.59
13	2-Phenyl-2-propanol	-0.57
14	3-Amono-2-cresol	-0.55
15	2,4,6-*tris*-(Dimethylaminomethyl)phenol	-0.52
16	4-Methylbenzyl alcohol	-0.49
17	Phenylacetic acid hydrazide	-0.48
18	3-Cyanoaniline	-0.47
19	Acetophenone	-0.46
20	2-Methylbenzyl alcohol	-0.43
21	(±)1-Phenyl-1-propanol	-0.43
22	2,3-Dimethylaniline	-0.43
23	2,6-Dimethylaniline	-0.43
24	2-Methyl-1-phenyl-2-propanol	-0.41
25	*N*-Methylphenethylamine	-0.41
26	2-Phenyl-1-propanol	-0.4
27	3-Fluorobenzyl alcohol	-0.39
28	4-Hydroxybenzyl cyanide	-0.38
29	4-Cyanobenzamide	-0.38
30	2-Fluoroaniline	-0.37
31	3,5-Dimethylaniline	-0.36
32	Benzyl cyanide	-0.36
33	Phenol	-0.35
34	3-Methoxyphenol	-0.33
35	2,5-Dimethylaniline	-0.33
36	2-Methylphenol	-0.29
37	2,4-Dimethylaniline	-0.29
38	3-Methylaniline	-0.28
39	β- Methylphenethylamine	-0.28
40	4-Methylphenethyl alcohol	-0.26
41	Benzylamine	-0.24
42	2-Tolunitrile	-0.24
43	3-Methylbenzyl alcohol	-0.24
44	Aniline	-0.23
45	2-Ethylaniline	-0.22
46	3-Nitrobenzyl alcohol	-0.22
47	3-Phenyl-1-propanol	-0.21
48	Benzaldehyde	-0.2
49	2-Phenyl-3-butyn-2-ol	-0.18
50	1-Phenylethylamine	-0.18
51	2-Chloroaniline	-0.17
52	1-Phenyl-2-butanol	-0.16
53	3,4-Dimethylaniline	-0.16
54	2-Methylaniline	-0.16
55	4-Methylphenol	-0.16
56	3-Phenylpropionitrile	-0.16
57	3-Acetamidophenol	-0.16
58	4-Methoxyphenol	-0.14
59	Phenetole	-0.14
60	3-Hydroxy-4-methoxybenzaldehyde	-0.14
61	Chlorobenzene	-0.13
62	Benzene	-0.12
63	2-Phenyl-1-butanol	-0.11
64	Benzaldoxime	-0.11
65	Anisole	-0.1
66	3-Fluoroaniline	-0.1
67	2,4,5-Trimethoxybenzaldehyde	-0.1
68	(S±)-1-Phenyl-1-butanol	-0.09
69	3,5-Dimethoxyphenol	-0.09
70	3-Methylphenol	-0.08
71	3-Phenyl-2-propen-1-ol	-0.08
72	α,α-Dimethylbenzenepropanol	-0.07
73	Propiophenone	-0.07
74	2-Nitroanisole	-0.07
75	4-Methylaniline	-0.05
76	2,4,6-Trimethylaniline	-0.05
77	2-(4-Tolyl)-ethylamine	-0.04
78	3-Ethylaniline	-0.03
79	3-Methoxy-4-hydroxybenzaldehyde	-0.03
80	4-Hydroxy-3-methoxybenzonitrile	-0.03
81	Ethyl phenylcyanoacetate	-0.02
82	(*R*±)-1-Phenyl-1-butanol	-0.01
83	4-Methylbenzylamine	-0.01
84	Thioacetanilide	-0.01
85	3-Phenyl-1-butanol	0.01
86	α-Methylbenzyl cyanide	0.01
87	4-Ethoxyphenol	0.01
88	3-Ethoxy-4-hydroxybenzaldehyde	0.02
89	4-Fluorophenol	0.02
90	4-Ethylaniline	0.03
91	3-Nitroaniline	0.03
92	4-Chloroaniline	0.05
93	(±)-2-Phenyl-2-butanol	0.06
94	Benzyl chloride	0.06
95	*N*-Methylaniline	0.06
96	4-Ethylbenzyl alcohol	0.07
97	*N*-Ethylaniline	0.07
98	Bromobenzene	0.08
99	2-Nitroaniline	0.08
100	2-Propylaniline	0.08
101	3-Hydroxybenzaldehyde	0.08
102	Thiobenzamide	0.09
103	1-Fluoro-4-nitrobenzene	0.1
104	2-Bromobenzyl alcohol	0.1
105	4-Methoxybenzonitrile	0.1
106	3,5-Dimethylphenol	0.11
107	3-Nitrobenzaldehyde	0.11
108	4-Phenyl-1-butanol	0.12
109	4^/^-Hydroxypropiophenone	0.12
110	2-*iso*-Propylaniline	0.12
111	3,4-Dimethylphenol	0.12
112	2,3-Dimethylphenol	0.12
113	4-Chlororesorcinol	0.13
114	2,4-Dimethylphenol	0.14
115	2-(4-Chlorophenyl)-ethylamine	0.14
116	Nitrobenzene	0.14
117	2,5-Dimethylphenol	0.14
118	4-Phenylbutyronitrile	0.15
119	3-Chlorobenzyl alcohol	0.15
120	2-Anisaldehyde	0.15
121	2-Ethylphenol	0.16
122	4-Chlorobenzylamine	0.16
123	(±)-1-Phenyl-2-pentanol	0.16
124	Cinnamonitrile	0.16
125	2-Nitrobenzaldehyde	0.17
126	Thioanisole	0.18
127	2-Chloro-4-methylaniline	0.18
128	4-*iso*-Propylbenzyl alcohol	0.18
129	Phenyl-1,3-dialdehyde	0.18
130	2-Fluorophenol	0.19
131	4-Nitrobenzaldehyde	0.2
132	4-Ethylphenol	0.21
133	Butyrophenone	0.21
134	4-*iso*-propylaniline	0.22
135	3-Chloroaniline	0.22
136	4-(Dimethylamino)-benzaldehyde	0.23
137	3-Anisaldehyde	0.23
138	1-Fluoro-2-nitrobenzene	0.23
139	4-Xylene	0.25
140	Toluene	0.25
141	4-Methylanisole	0.25
142	4-Chlorobenzyl alcohol	0.25
143	2,4-Dihydroxyacetophenone	0.25
144	2-Nitrotoluene	0.26
145	Pentafluoroaniline	0.26
146	2-Phenylpyridine	0.27
147	3-Hydroxy-4-nitrobenzaldehyde	0.27
148	2,3,6-Trimethylphenol	0.28
149	3-Ethylphenol	0.29
150	2,6-Diethylaniline	0.31
151	Methyl-4-methylaminobenzoate	0.31
152	Benzoyl cyanide	0.31
153	4-Chlorophenethyl alcohol	0.32
154	3^/^-Nitroacetophenone	0.32
155	2-Allylphenol	0.33
156	5-Hydroxy-2-nitrobenzaldehyde	0.33
157	2-Bromophenol	0.33
158	2,5-Difluoronitrobenzene	0.33
159	4-Chloro-2-methylaniline	0.35
160	2-Iodoaniline	0.35
161	2,3,5-trimethylphenol	0.36
162	Iodobenzene	0.36
163	4-(*tert*)-Butylaniline	0.36
164	4-methyl-2-nitroaniline	0.37
165	2-Amino-4-(*tert*)-butylphenol	0.37
166	2-Benzylpyridine	0.38
167	3-Chloro-2-methylaniline	0.38
168	3-Chloro-4-methylaniline	0.39
169	Methyl-4-nitrobenzoate	0.39
170	4-Chlorobenzaldehyde	0.4
171	5-Phenyl-1-pentanol	0.42
172	(2-Bromoethyl)-benzene	0.42
173	2,4,6-Trimethylphenol	0.42
174	3-Nitrotoluene	0.42
175	2-Hydroxybenzaldehyde	0.42
176	1-Chloro-4-nitrobenzene	0.43
177	Dimethylnitroterephthalate	0.43
178	2-Amino-5-chlorobenzonitrile	0.44
179	3-Nitrobenzonitrile	0.45
180	4-Bromotoluene	0.47
181	3-Phenylpyridine	0.47
182	4-*iso*-Propylphenol	0.47
183	4-(*tert*)-Butylbenzyl alcohol	0.48
184	Benzhydrol	0.5
185	5-Chloro-2-methylaniline	0.5
186	3-Nitrophenol	0.51
187	1,2-Dichlorobenzene	0.53
188	2-Chloro-5-nitrobenzaldehyde	0.53
189	4-Chlorophenol	0.54
190	Phenyl propargyl sulfide	0.54
191	2-Chloro-5-methylphenol	0.54
192	2-Hydroxy-4-methoxyacetophenone	0.55
193	2,4-Dichloroaniline	0.56
194	1,2-Dimethyl-3-nitrobenzene	0.56
195	Valerophenone	0.56
196	4-Methyl-2-nitrophenol	0.57
197	2,5-Dichloroaniline	0.58
198	*trans*-Methyl cinnamate	0.58
199	1,2-Dimethyl-4-nitrobenzene	0.59
200	5-Chloro-2-hydroxybenzamide	0.59
201	5-Methyl-2-nitrophenol	0.59
202	4-Chloroanisole	0.6
203	2-Bromo-4-methylphenol	0.6
204	4-Bromophenyl acetonitrile	0.6
205	4-Butoxyaniline	0.61
206	4-*sec*-Butylaniline	0.61
207	3-*iso*-Propylphenol	0.61
208	2-*iso*-Propylphenol	0.61
209	3-Methyl-2-nitrophenol	0.61
210	4-Hydroxy-3-nitrobenzaldehyde	0.61
211	5-Bromovanillin	0.62
212	α,α,α-Trifluoro-4-cresol	0.62
213	4-Benzylpyridine	0.63
214	4-Propylphenol	0.64
215	Benzylidine malononitrile	0.64
216	4-Nitrotoluene	0.65
217	3-Iodoaniline	0.65
218	Benzyl methacrylate	0.65
219	4-Chlorobenzylcyanide	0.66
220	2-Methyl-5-nitrophenol	0.66
221	2-Nitroresorcinol	0.66
222	1-Bromo-4-ethylbenzene	0.67
223	4-*iso*-Propylbenzaldehyde	0.67
224	2-Nitrophenol	0.67
225	1,4-Dibromobenzene	0.68
226	2-Chloro-6-nitrotoluene	0.68
227	1-Chloro-2-nitrobenzene	0.68
228	4-Bromophenol	0.68
229	4-Benzoylaniline	0.68
230	*iso*-Propylbenzene	0.69
231	2-Chloro-4,5-dimethylphenol	0.69
232	4-Butoxyphenol	0.7
233	4-Chloro-2-methylphenol	0.7
234	3,5-Dichloroaniline	0.71
235	2-Hydroxy-4,5-dimethylacetophenone	0.71
236	Ethyl-4-nitrobenzoate	0.71
237	3-Nitroanisole	0.72
238	2,4-Dinitroaniline	0.72
239	1-Chloro-3-nitrobenzene	0.73
240	2,6-Dichlorophenol	0.73
241	3-*tert*-Butylphenol	0.74
242	1,1-Diphenyl-2-propanol	0.75
243	2-Chloro-4-nitroaniline	0.75
244	1-Bromo-2-nitrobenzene	0.75
245	2-Methoxy-4-propenylphenol	0.75
246	2-Chloromethyl-4-nitrophenol	0.75
247	4,5-Difluoro-2-nitroaniline	0.75
248	2,6-Diisopropylaniline	0.76
249	3-Chloro-5-methoxyphenol	0.76
250	4-Ethoxy-2-nitroaniline	0.76
251	1,3-Dinitrobenzene	0.76
252	α,α,α-4-Tetrafluoro-3-touidine	0.77
253	Ethyl-4-methoxybenzoate	0.77
254	(±)-1,2-Diphenyl-2-propanol	0.8
255	4-Chloro-3-methylphenol	0.8
256	3-Chloro-4-fluoronitrobenzene	0.8
257	Methyl-2,5-dichlorobenzoate	0.81
258	4-Chloro-2-nitrotoluene	0.82
259	Pentafluorobenzaldehyde	0.82
260	4-Bromophenyl-3-pyridyl ketone	0.82
261	Methyl-4-chloro-2-nitrobenzoate	0.82
262	4-Nitrophenetole	0.83
263	2,6-Dinitrophenol	0.83
264	2,6-Dinitroaniline	0.84
265	4-Iodophenol	0.85
266	1,3,5-Trimethyl-2-nitrobenzene	0.86
267	6-Phenyl-1-hexanol	0.87
268	3-Chlorophenol	0.87
269	Benzophenone	0.87
270	1,3,5-Trichlorobenzene	0.87
271	2,4-Dinitrotoluene	0.87
272	4-(*tert*)-Butylphenol	0.91
273	4-Biphenylmethanol	0.92
274	3,4,5-Trimethylphenol	0.93
275	2,2^/^,4,4^/^-Tetrahydroxybenzophenone	0.96
276	4-Pentyloxyaniline	0.97
277	2,4-Dichloronitrobenzene	0.99
278	(*trans*)-Ethyl cinnamate	0.99
279	4-Benzoylphenol	1.02
280	1-Bromo-3-nitrobenzene	1.03
281	2,4-Dichlorophenol	1.04
282	2,5-Dinitrophenol	1.04
283	2,4-Dichlorobenzaldehyde	1.04
284	Biphenyl	1.05
285	2,4-Dinitrophenol	1.06
286	4-Butylaniline	1.07
287	3,4-Dichlorotoluene	1.07
288	2,3-Dichloronitrobenzene	1.07
289	Benzyl-4-hydroxylphenyl ketone	1.07
290	1,2,4-Trichlorobenzene	1.08
291	4-Chloro-3-ethylphenol	1.08
292	1-Fluoro-3-iodo-5-nitrobenzene	1.09
293	Resorcinol monobenzoate	1.11
294	6-Chloro-2,4-dinitroaniline	1.12
295	4-Biphenylcarboxaldehyde	1.12
296	3,5-Dichloronitrobenzene	1.13
297	2,5-Dichloronitrobenzene	1.13
298	2-Bromo-5-nitrotoluene	1.16
299	3,4-Dichloronitrobenzene	1.16
300	6-*tert*-butyl-2,4-dimethylphenol	1.16
301	4-Bromo-2,6-dimethylphenol	1.16
302	2,2^/^-Dihydroxybenzophenone	1.16
303	3,5-Dibromo-4-hydroxybenzonitrile	1.16
304	4-(Pentyloxy)-benzaldehyde	1.18
305	4-Nitrobenzyl chloride	1.18
306	Hexanophenone	1.19
307	4-Chloro-3,5-dimethylphenol	1.2
308	4-*tert*-Pentylphenol	1.23
309	*n*-Propyl cinnamate	1.23
310	2-Bromo-4,6-dinitroaniline	1.24
311	*n*-Butylbenzene	1.25
312	1,2-Dinitrobenzene	1.25
313	4-Bromobenzophenone	1.26
314	2,4-Dichloro-6-nitroaniline	1.26
315	4-Phenoxybenzaldehyde	1.26
316	4-Chloro-3-nitrophenol	1.27
317	4-Bromo-6-chloro-2-cresol	1.28
318	2,4,5-Trichloroaniline	1.3
319	1,4-Dinitrobenzene	1.3
320	2-Nitrobiphenyl	1.3
321	5-Pentylresorcinol	1.31
322	Ethyl-4-bromobenzoate	1.33
323	2^/^,3^/^,4^/^-Trichloroacetophenone	1.34
324	Phenyl benzoate	1.35
325	Phenyl-4-hydroxybenzoate	1.37
326	2,5-Dibromonitrobenzene	1.37
327	4-Hexyloxyaniline	1.38
328	2,4-Dibromophenol	1.4
329	2,4,6-Trichlorophenol	1.41
330	Phenyl isothiocyanate	1.41
331	2-Hydroxy-4-methoxybenzophenone	1.42
332	1,3,5-Trichloro-2-nitrobenzene	1.43
333	Benzyl benzoate	1.45
334	*iso*-Amyl-4-hydroxybenzoate	1.48
335	2,5-Diphenyl-1,4-benzoquinone	1.48
336	4-Chlorobenzophenone	1.5
337	1,2,3-Trichloro-4-nitrobenzene	1.51
338	1,2,4-Trichloro-5-nitrobenzene	1.53
339	*n*-Butyl cinnamate	1.53
340	3-Chlorobenzophenone	1.55
341	3,5-Dichlorosalicylaldehyde	1.55
342	Heptanophenone	1.56
343	3,5-Dichlorophenol	1.56
344	4-Nitrophenyl phenyl ether	1.58
345	2,4-Dibromo-6-nitroaniline	1.62
346	4-Chloro-6-nitro-3-cresol	1.63
347	Pentafluorophenol	1.63
348	3,5-Di-*tert*-butylphenol	1.64
349	3,5-Dibromosalicylaldehyde	1.65
350	3-Trifluoromethyl-4-nitrophenol	1.65
351	4,5-Dichloro-2-nitroaniline	1.66
352	2,4-Dinitro-1-fluorobenzene	1.71
353	2-(Benzylthio)-3-nitropyridine	1.72
354	4,6-Dinitro-2-methylphenol	1.73
355	2,4-Dichloro-6-nitrophenol	1.75
356	2,3,5,6-Tetrachloroaniline	1.76
357	4-Bromo-2,6-dichlorophenol	1.78
358	2,3,4,5-Tetrachloronitrobenzene	1.78
359	*n* -Amylbenzene	1.79
360	4-Hexylresorcinol	1.8
361	4-(*tert*)-Butyl-2,6-dinitrophenol	1.8
362	2,6-Diiodo-4-nitrophenol	1.81
363	2,3,5,6- Tetrachloronitrobenzene	1.82
364	2,3,4,6- Tetrachloronitrobenzene	1.87
365	Octanophenone	1.89
366	1,2,3-Trifluoro-4-nitrobenzene	1.89
367	2,4,6-Tribromophenol	1.91
368	2,3,4,5-Tetrachloroaniline	1.96
369	4-Ethylbiphenyl	1.97
370	1,2,4,5-Tetrachlorobenzene	2
371	Pentachlorophenol	2.07
372	2,4,5-Trichlorophenol	2.1
373	2,4-Dinitro-1-iodobenzene	2.12
374	1-Chloro-2,4-dinitrobenzene	2.16
375	2,3,4,6-Tetrachlorophenol	2.18
376	1,3,5-Trichloro-2,4-dinitrobenzene hemihydrate	2.19
377	1,2-Dichloro-4,5-dinitrobenzene	2.21
378	1,5-Dichloro-2,3-dinitrobenzene	2.42
379	Nonylphenol	2.47
380	3,4,5,6-Tetrabromo-2-cresol	2.57
381	1,3-Dinitro-2,4,5-trichlorobenzene	2.60
382	Pentabromophenol	2.66
383	2,3,4,5-Tetrachlorophenol	2.72
384	1,4-Dinitrotetrachlorobenzene	2.82

**Table 4:** Comparison of statistical qualities and validation parameters of different models (Data set I).

Trial No.	No. of predictor variables	LOF	R^2^	Q^2^	R^2^_pred_	r_m_^2^_(LOO)_	r_m_^2^_(test)_	r_m_^2^ (overall)	r_m_^2^_(overall)_(adjusted)	R_r_^2^	R_p_^2^
01	4	0.276	0.677	0.642	0.329	0.466	0.313	0.444	0.418	0.143	0.495
02	2	0.336	0.596	0.569	0.358	0.378	0.348	0.383	0.369	0.098	0.421
**03**	**3**	**0.380**	**0.552**	**0.511**	0.595*	**0.367**	**0.566**	**0.400**	**0.379**	**0.118**	**0.364**
04	4	0.349	0.621	0.569	0.438	0.409	0.391	0.407	0.379	0.108	0.445
05	4	0.323	0.634	0.567	0.367	0.407	0.339	0.402	0.374	0.078	0.473
06	2	0.357	0.542	0.511	0.542	0.366	0.508	0.399	0.385	0.116	0.354
07	3	0.351	0.597	0.560	0.436	0.402	0.466	0.416	0.395	0.100	0.421
08	4	0.304	0.660	0.620	0.346	0.414	0.303	0.400	0.371	0.117	0.486
09	3	0.256	0.697	0.675	0.080	0.494	0.142	0.417	0.396	0.102	0.538
10	4	0.347	0.596	0.549	0.530	0.394	0.509	0.431	0.404	0.087	0.425
11	3	0.359	0.567	0.519	0.556	0.372	0.506	0.407	0.386	0.102	0.387
12	3	0.294	0.663	0.632	0.117	0.458	0.133	0.405	0.384	0.138	0.480
13	4	0.273	0.678	0.640	0.326	0.463	0.324	0.441	0.414	0.089	0.520
14	3	0.345	0.604	0.568	0.390	0.408	0.364	0.410	0.389	0.176	0.395
15	3	0.369	0.558	0.502	0.523	0.360	0.500	0.386	0.364	0.130	0.365
16	3	0.318	0.627	0.584	0.282	0.401	0.310	0.373	0.351	0.126	0.444
17	4	0.330	0.622	0.562	0.462	0.405	0.445	0.417	0.389	0.100	0.449
18	4	0.370	0.581	0.531	0.542	0.381	0.529	0.415	0.387	0.091	0.407
19	4	0.346	0.615	0.564	0.447	0.406	0.427	0.411	0.383	0.111	0.437
20	3	0.289	0.657	0.625	0.301	0.452	0.268	0.420	0.400	0.108	0.487
21	3	0.299	0.648	0.614	0.254	0.443	0.241	0.412	0.391	0.124	0.469
22	3	0.324	0.610	0.573	0.426	0.410	0.426	0.418	0.397	0.143	0.417
23	3	0.347	0.581	0.519	0.471	0.373	0.440	0.398	0.377	0.116	0.396
24	4	0.290	0.673	0.636	0.238	0.461	0.254	0.425	0.398	0.092	0.513
25	3	0.313	0.622	0.591	0.343	0.425	0.324	0.411	0.390	0.122	0.440
26	4	0.257	0.686	0.645	0.233	0.467	0.179	0.405	0.377	0.108	0.521
27	4	0.299	0.659	0.615	0.212	0.445	0.219	0.404	0.376	0.095	0.495
28	4	0.342	0.603	0.558	0.497	0.369	0.468	0.396	0.367	0.122	0.418
29	4	0.385	0.593	0.536	0.544	0.386	0.474	0.399	0.370	0.118	0.409
30	4	0.324	0.627	0.580	0.394	0.418	0.361	0.414	0.386	0.095	0.457
31	4	0.353	0.592	0.544	0.286	0.389	0.260	0.368	0.338	0.111	0.411
32	3	0.314	0.636	0.602	0.264	0.434	0.272	0.411	0.390	0.113	0.460
33	5	0.295	0.685	0.644	0.179	0.468	0.201	0.413	0.378	0.106	0.521
34	2	0.271	0.652	0.629	0.263	0.454	0.244	0.415	0.401	0.132	0.470
35	4	0.340	0.615	0.566	0.303	0.406	0.273	0.387	0.358	0.088	0.447
36	4	0.335	0.641	0.604	0.286	0.436	0.321	0.425	0.398	0.096	0.473
37	3	0.341	0.585	0.547	0.517	0.392	0.503	0.413	0.392	0.095	0.409
38	3	0.279	0.659	0.628	0.253	0.454	0.266	0.419	0.398	0.166	0.463
**39**	**4**	**0.210**	**0.731**	0.701*	**0.240**	**0.517**	**0.285**	0.452*	**0.426**	**0.135**	**0.564**
40	3	0.302	0.630	0.597	0.359	0.429	0.367	0.422	0.402	0.158	0.433
41	3	0.380	0.558	0.510	0.565	0.367	0.542	0.399	0.378	0.107	0.375
**42**	**3**	**0.404**	**0.557**	**0.517**	0.595*	**0.374**	**0.578**	**0.403**	**0.382**	**0.106**	**0.374**
43	3	0.285	0.632	0.585	0.284	0.420	0.282	0.396	0.375	0.134	0.446
44	4	0.337	0.611	0.565	0.424	0.405	0.453	0.421	0.393	0.137	0.421
45	3	0.360	0.602	0.567	0.320	0.408	0.299	0.398	0.377	0.094	0.429
46	6	0.302	0.697	0.646	0.239	0.471	0.262	0.431	0.389	0.076	0.549
47	3	0.312	0.615	0.573	0.369	0.411	0.365	0.411	0.390	0.134	0.427
48	3	0.298	0.653	0.617	0.167	0.446	0.179	0.404	0.383	0.134	0.470
49	3	0.290	0.623	0.589	0.400	0.421	0.412	0.424	0.404	0.097	0.452
50	2	0.311	0.590	0.561	0.420	0.428	0.401	0.415	0.401	0.106	0.410

*Models with maximum Q^2^, R^2^_pred_ and r_m_^2^_(overall)_ values are shown in bold.

**Table 5:** Comparison of statistical qualities and validation parameters of different models (Data set II).

Trial No.	No. of predictor variables	LOF	R^2^	Q^2^	R^2^_pred_	r_m_^2^_(LOO)_	r_m_^2^_(test)_	r_m_^2^_(overall)_	r_m_^2^_(overall)_(adjusted)	R_r_^2^	R_p_^2^
01	4	1.306	0.673	0.617	0.325	0.462	0.280	0.426	0.390	0.076	0.520
02	4	1.696	0.577	0.510	0.479	0.384	0.433	0.393	0.354	0.078	0.408
03	4	1.529	0.612	0.559	0.347	0.418	0.326	0.408	0.370	0.078	0.447
04	6	1.620	0.607	0.517	0.540	0.385	0.473	0.415	0.357	0.079	0.441
05	4	1.347	0.646	0.606	0.441	0.449	0.430	0.444	0.409	0.071	0.490
06	4	1.534	0.606	0.548	0.600	0.408	0.585	0.437	0.401	0.059	0.448
07	4	1.496	0.642	0.585	0.024	0.440	0.149	0.372	0.332	0.107	0.470
**08**	**4**	**1.644**	**0.553**	**0.468**	0.714*	**0.357**	**0.684**	**0.408**	**0.370**	**0.050**	**0.392**
09	4	1.593	0.588	0.521	0.633	0.391	0.535	0.423	0.386	0.066	0.425
10	2	1.514	0.547	0.513	0.325	0.381	0.291	0.367	0.348	0.104	0.364
11	5	1.457	0.658	0.589	0.448	0.439	0.472	0.448	0.403	0.051	0.513
12	4	1.436	0.642	0.596	0.470	0.443	0.435	0.439	0.403	0.075	0.483
13	4	1.517	0.590	0.529	0.613	0.394	0.577	0.433	0.397	0.074	0.424
14	4	1.318	0.654	0.609	0.443	0.452	0.433	0.449	0.414	0.076	0.497
15	4	1.523	0.586	0.523	0.652	0.390	0.573	0.434	0.398	0.103	0.407
16	4	1.466	0.622	0.567	0.203	0.422	0.243	0.397	0.359	0.094	0.452
17	6	1.409	0.681	0.613	0.597	0.457	0.597	0.471	0.419	0.072	0.531
18	5	1.253	0.705	0.656	0.351	0.493	0.328	0.448	0.403	0.072	0.561
19	5	1.173	0.711	0.665	0.331	0.499	0.312	0.455	0.411	0.100	0.556
20	5	1.546	0.630	0.558	0.507	0.416	0.468	0.425	0.379	0.060	0.476
21	4	1.288	0.681	0.636	-0.028	0.477	0.129	0.382	0.343	0.056	0.538
**22**	**6**	**1.349**	**0.675**	**0.612**	**0.608**	**0.457**	**0.538**	0.488*	**0.438**	**0.077**	**0.522**
23	5	1.392	0.660	0.600	0.488	0.449	0.467	0.447	0.402	0.046	0.517
24	5	1.321	0.680	0.637	0.409	0.475	0.374	0.451	0.407	0.086	0.524
25	6	1.360	0.701	0.635	0.525	0.476	0.484	0.475	0.423	0.075	0.555
26	6	1.231	0.722	0.666	0.403	0.504	0.363	0.464	0.411	0.068	0.584
27	4	1.116	0.708	0.672	0.282	0.503	0.254	0.451	0.416	0.063	0.569
28	5	1.363	0.648	0.582	0.588	0.432	0.552	0.455	0.411	0.097	0.481
29	5	1.414	0.627	0.564	0.614	0.418	0.572	0.447	0.402	0.110	0.451
30	4	1.267	0.673	0.630	0.213	0.470	0.260	0.436	0.400	0.058	0.528
31	4	1.454	0.626	0.577	0.330	0.430	0.302	0.411	0.374	0.084	0.461
32	5	1.595	0.613	0.540	0.433	0.407	0.349	0.391	0.342	0.081	0.447
33	4	1.408	0.633	0.577	0.249	0.429	0.248	0.392	0.353	0.068	0.476
34	4	1.522	0.586	0.517	0.656	0.387	0.635	0.434	0.398	0.070	0.421
35	6	1.075	0.758	0.704	-0.002	0.536	0.108	0.422	0.365	0.083	0.623
36	4	1.446	0.598	0.535	0.616	0.398	0.545	0.445	0.410	0.074	0.433
37	4	1.695	0.552	0.486	0.614	0.368	0.559	0.409	0.371	0.098	0.372
38	4	1.305	0.650	0.596	0.368	0.442	0.450	0.443	0.408	0.080	0.491
39	5	1.298	0.687	0.616	0.361	0.463	0.322	0.437	0.392	0.090	0.531
40	4	1.330	0.663	0.617	0.125	0.460	0.149	0.397	0.359	0.078	0.507
41	5	1.319	0.682	0.620	0.077	0.465	0.140	0.393	0.344	0.093	0.523
42	4	1.601	0.556	0.485	0.656	0.365	0.634	0.413	0.376	0.047	0.396
43	4	1.218	0.651	0.588	0.496	0.436	0.482	0.444	0.409	0.060	0.500
**44**	**6**	**0.993**	**0.770**	0.723*	**0.136**	**0.551**	**0.169**	**0.462**	**0.409**	**0.075**	**0.642**
45	4	1.097	0.705	0.663	0.200	0.496	0.173	0.427	0.391	0.078	0.558
46	5	1.494	0.633	0.558	0.636	0.418	0.550	0.439	0.394	0.103	0.461
47	5	1.392	0.649	0.575	0.545	0.427	0.536	0.439	0.394	0.059	0.498
48	5	1.254	0.682	0.623	0.077	0.466	0.134	0.388	0.339	0.070	0.533
49	4	1.252	0.684	0.636	0.151	0.476	0.173	0.411	0.374	0.073	0.535
50	5	1.270	0.657	0.583	0.556	0.433	0.548	0.447	0.402	0.057	0.509

*Models with maximum Q^2^, R^2^_pred_ and r_m_^2^_(overall)_ values are shown in bold.

**Table 6:** Comparison of statistical qualities and validation parameters of different models (Data set III).

Trial No.	No. of predictor variables	LOF	R^2^	Q^2^	R^2^_pred_	r_m_^2^_(LOO)_	r_m_^2^_(test)_	r_m_^2^_(overall)_	r_m_^2^_(overall)_(adjusted)	R_r_^2^	R_p_^2^
01	08	0.132	0.774	0.758	0.551	0.711	0.559	0.675	0.666	0.042	0.662
02	08	0.147	0.753	0.721	0.641	0.694	0.647	0.693	0.684	0.037	0.637
03	08	0.167	0.721	0.660	0.750	0.668	0.721	0.657	0.647	0.025	0.601
04	07	0.139	0.764	0.744	0.685	0.723	0.586	0.667	0.659	0.045	0.648
05	06	0.135	0.760	0.671	0.681	0.659	0.653	0.631	0.623	0.052	0.640
06	07	0.148	0.747	0.727	0.703	0.704	0.661	0.680	0.672	0.037	0.629
07	06	0.159	0.731	0.708	0.612	0.694	0.620	0.669	0.662	0.035	0.610
08	07	0.144	0.758	0.703	0.641	0.681	0.628	0.650	0.641	0.031	0.646
09	07	0.123	0.772	0.759	0.572	0.712	0.577	0.680	0.672	0.036	0.662
10	09	0.137	0.765	0.734	0.742	0.701	0.752	0.677	0.667	0.042	0.651
11	09	0.145	0.748	0.713	0.583	0.693	0.590	0.657	0.646	0.036	0.631
12	08	0.150	0.738	0.672	0.734	0.669	0.712	0.669	0.660	0.037	0.618
13	12	0.129	0.780	0.738	0.716	0.698	0.669	0.691	0.678	0.032	0.675
14	09	0.143	0.759	0.703	0.622	0.679	0.595	0.639	0.627	0.038	0.645
15	09	0.122	0.789	0.769	0.545	0.724	0.518	0.658	0.647	0.029	0.688
16	07	0.149	0.734	0.692	0.753	0.676	0.728	0.688	0.680	0.032	0.615
17	07	0.123	0.770	0.755	0.595	0.706	0.594	0.672	0.664	0.037	0.659
18	09	0.138	0.756	0.731	0.741	0.699	0.671	0.688	0.678	0.025	0.646
19	07	0.162	0.726	0.676	0.678	0.673	0.674	0.643	0.634	0.027	0.607
20	07	0.138	0.769	0.752	0.577	0.720	0.536	0.659	0.650	0.028	0.662
21	08	0.147	0.733	0.690	0.731	0.669	0.643	0.670	0.661	0.047	0.607
22	08	0.160	0.730	0.693	0.731	0.679	0.688	0.666	0.656	0.044	0.605
23	06	0.131	0.769	0.755	0.497	0.710	0.478	0.654	0.647	0.035	0.659
24	09	0.154	0.751	0.721	0.635	0.697	0.610	0.676	0.666	0.038	0.634
25	06	0.108	0.784	0.772	0.575	0.715	0.594	0.674	0.667	0.023	0.684
26	08	0.153	0.723	0.697	0.781	0.683	0.752	0.688	0.679	0.032	0.601
27	08	0.158	0.732	0.706	0.744	0.692	0.742	0.687	0.678	0.025	0.615
28	08	0.164	0.726	0.696	0.736	0.696	0.686	0.664	0.654	0.052	0.596
29	07	0.165	0.720	0.690	0.746	0.681	0.727	0.683	0.675	0.038	0.594
**30**	**09**	**0.123**	**0.792**	**0.771**	**0.692**	**0.720**	**0.687**	0.699*	**0.689**	**0.052**	**0.682**
31	08	0.118	0.783	0.766	0.580	0.716	0.559	0.665	0.655	0.032	0.678
32	07	0.162	0.709	0.685	0.712	0.679	0.678	0.681	0.673	0.040	0.580
33	09	0.144	0.759	0.730	0.730	0.705	0.699	0.683	0.673	0.034	0.646
34	13	0.154	0.758	0.718	0.678	0.699	0.638	0.674	0.659	0.025	0.649
35	13	0.130	0.795	0.757	0.704	0.715	0.701	0.681	0.666	0.033	0.694
36	08	0.146	0.754	0.728	0.579	0.703	0.510	0.641	0.631	0.035	0.639
37	05	0.135	0.769	0.757	0.382	0.720	0.385	0.646	0.640	0.032	0.660
38	10	0.151	0.748	0.719	0.601	0.693	0.568	0.659	0.647	0.033	0.632
39	06	0.164	0.709	0.687	0.739	0.681	0.714	0.673	0.666	0.034	0.583
40	08	0.153	0.739	0.710	0.758	0.692	0.722	0.691	0.682	0.037	0.619
41	08	0.164	0.727	0.692	0.680	0.684	0.664	0.659	0.649	0.032	0.606
42	09	0.139	0.766	0.734	0.522	0.697	0.473	0.634	0.622	0.036	0.655
43	07	0.147	0.748	0.727	0.643	0.699	0.638	0.661	0.653	0.039	0.630
44	08	0.167	0.726	0.699	0.656	0.684	0.600	0.655	0.645	0.031	0.605
**45**	**07**	**0.168**	**0.700**	**0.677**	0.834*	**0.676**	**0.753**	**0.685**	**0.677**	**0.027**	**0.574**
46	08	0.162	0.708	0.676	0.753	0.679	0.725	0.676	0.667	0.039	0.579
47	07	0.151	0.736	0.712	0.659	0.689	0.669	0.674	0.666	0.042	0.613
48	07	0.159	0.723	0.695	0.737	0.685	0.714	0.685	0.677	0.021	0.606
49	08	0.130	0.781	0.764	0.596	0.719	0.610	0.693	0.684	0.035	0.675
**50**	**09**	**0.123**	**0.792**	0.774*	**0.596**	**0.726**	**0.587**	**0.678**	**0.668**	**0.023**	**0.695**

*Models with maximum Q^2^, R^2^_pred_ and r_m_^2^_(overall)_ values are shown in bold.

## Supplementary Files

Supplementary File 1
