# Roy *et al*. On Two Novel Parameters for Validation of Predictive QSAR Models. *Molecules*, 2009, *14*, 1660-1701

**DOI:** 10.3390/molecules15010604

**Published:** 2010-01-26

**Authors:** Partha Pratim Roy, Somnath Paul, Indrani Mitra, Kunal Roy

**Affiliations:** Drug Theoretics and Cheminformatics Lab, Division of Medicinal and Pharmaceutical Chemistry, Department of Pharmaceutical Technology, Jadavpur University, Kolkata 700 032, India; E-Mails: partha_chemju@yahoo.co.in (P.P.R.); somnath_juph@yahoo.co.in (S.P.); indranimitra06@gmail.com (I.M.)

The authors wish to make the following corrections to this paper [1]:

**Abstract:** The sentence “The parameter r_m_^2^_(overall)_ penalizes a model for large differences between observed and predicted values of the compounds of the whole set (considering both training and test sets) while the parameter R_p_^2^ penalizes model R^2^ for large differences between determination coefficient of nonrandom model and square of mean correlation coefficient of random models in case of a randomization test.” should read as: The parameter r_m_^2^_(overall)_ penalizes a model for large differences between observed and predicted values of the compounds of the whole set (considering both training and test sets) while the parameter R_p_^2^ penalizes model R^2^ for a small difference between determination coefficient of nonrandom model and square of mean correlation coefficient of random models in case of a randomization test.

**Section 2.3.2.4:** The sentence “We have used a parameter R_p_^2^ [32] in the present paper, which penalizes the model R^2^ for the difference between squared mean correlation coefficient (R_r_^2^) of randomized models and squared correlation coefficient (R^2^) of the non-randomized model.” should read as:

We have used a parameter R_p_^2^ [32] in the present paper, which penalizes the model R^2^ for a small difference between squared mean correlation coefficient (R_r_^2^) of randomized models and squared correlation coefficient (R^2^) of the non-randomized model.

**Conclusions:** The sentence “The parameter R_p_^2^ penalizes model R^2^ for large differences between determination coefficient of nonrandom model and square of mean correlation coefficient of random models in case of a randomization test and thus confirms whether a model has been obtained by chance or not.” should read as: The parameter R_p_^2^ penalizes model R^2^ for a small difference between determination coefficient of nonrandom model and square of mean correlation coefficient of random models in case of a randomization test and thus confirms whether a model has been obtained by chance or not.
