# Supplementary material for: On Two Novel Parameters for Validation of Predictive QSAR Models
Source: Molecules. 2009 Apr 29;14(5):1660–701. doi: 10.3390/molecules14051660 (PMC6254296; doi:10.3390/molecules14051660)
Supplement: Supplementary File 1 [file molecules-14-01660-s001.pdf]

Molecules **2010**, *15*, 604-605; doi:10.3390/molecules15010604

OPEN ACCESS

molecules

ISSN 1420-3049

www.mdpi.com/journal/molecules

Correction

Roy *et al*. On Two Novel Parameters for Validation of Predictive QSAR Models. *Molecules*, 2009, *14*, 1660-1701

Partha Pratim Roy, Somnath Paul, Indrani Mitra and Kunal Roy *

Drug Theoretics and Cheminformatics Lab, Division of Medicinal and Pharmaceutical Chemistry, Department of Pharmaceutical Technology, Jadavpur University, Kolkata 700 032, India;
E-Mails: partha_chemju@yahoo.co.in (P.P.R.); somnath_juph@yahoo.co.in (S.P.); indranimitra06@gmail.com (I.M.)

* Author to whom correspondence should be addressed; E-Mails: kunalroy_in@yahoo.com or kroy@pharma.jdvu.ac.in; Fax: +91-33-2837 1078.

Received: 25 January 2010 / Published: 26 January 2010

The authors wish to make the following corrections to this paper [1]:

Abstract: The sentence "The parameter $r_m^2{}_{(overall)}$ penalizes a model for large differences between observed and predicted values of the compounds of the whole set (considering both training and test sets) while the parameter R_p^2 penalizes model R^2 for large differences between determination coefficient of nonrandom model and square of mean correlation coefficient of random models in case of a randomization test." should read as: The parameter $r_m^2{}_{(overall)}$ penalizes a model for large differences between observed and predicted values of the compounds of the whole set (considering both training and test sets) while the parameter R_p^2 penalizes model R^2 for a small difference between determination coefficient of nonrandom model and square of mean correlation coefficient of random models in case of a randomization test.

Section 2.3.2.4: The sentence **"**We have used a parameter R_p^2 [32] in the present paper, which penalizes the model R^2 for the difference between squared mean correlation coefficient (R_r^2) of randomized models and squared correlation coefficient (R^2) of the non-randomized model." should read as: We have used a parameter R_p^2 [32] in the present paper, which penalizes the model R^2 for a small difference between squared mean correlation coefficient (R_r^2) of randomized models and squared correlation coefficient (R^2) of the non-randomized model.

Conclusions: The sentence "The parameter R_p^2 penalizes model R^2 for large differences between determination coefficient of nonrandom model and square of mean correlation coefficient of random models in case of a randomization test and thus confirms whether a model has been obtained by chance or not." should read as: The parameter R_p^2 penalizes model R^2 for a small difference between determination coefficient of nonrandom model and square of mean correlation coefficient of random models in case of a randomization test and thus confirms whether a model has been obtained by chance or not.

Reference

1. Roy, P.P.; Paul, S.; Mitra, I.; Roy, K. On Two Novel Parameters for Validation of Predictive QSAR Models. *Molecules* **2009**, *14*, 1660–1701.

© 2010 by the authors; licensee Molecular Diversity Preservation International, Basel, Switzerland. This article is an open-access article distributed under the terms and conditions of the Creative Commons Attribution license (http://creativecommons.org/licenses/by/3.0/).
